# Unfolding Simulations of Holomyoglobin from Four Mammals: Identification of Intermediates and β-Sheet Formation from Partially Unfolded States

**DOI:** 10.1371/journal.pone.0080308

**Published:** 2013-12-27

**Authors:** Pouria Dasmeh, Kasper P. Kepp

**Affiliations:** 1 Department of Chemistry, Technical University of Denmark, Kongens Lyngby, Denmark; 2 Max Planck Institute of Immunobiology and Epigenetics, Freiburg, Germany; Oak Ridge National Laboratory, United States of America

## Abstract

Myoglobin (Mb) is a centrally important, widely studied mammalian protein. While much work has investigated multi-step unfolding of apoMb using acid or denaturant, holomyoglobin unfolding is poorly understood despite its biological relevance. We present here the first systematic unfolding simulations of holoMb and the first comparative study of unfolding of protein orthologs from different species (sperm whale, pig, horse, and harbor seal). We also provide new interpretations of experimental mean molecular ellipticities of myoglobin intermediates, notably correcting for random coil and number of helices in intermediates. The simulated holoproteins at 310 K displayed structures and dynamics in agreement with crystal structures (*R*_g_ ∼1.48–1.51 nm, helicity ∼75%). At 400 K, heme was not lost, but some helix loss was observed in pig and horse, suggesting that these helices are less stable in terrestrial species. At 500 K, heme was lost within 1.0–3.7 ns. All four proteins displayed exponentially decaying helix structure within 20 ns. The C- and F-helices were lost quickly in all cases. Heme delayed helix loss, and sperm whale myoglobin exhibited highest retention of heme and D/E helices. Persistence of conformation (RMSD), secondary structure, and ellipticity between 2–11 ns was interpreted as intermediates of holoMb unfolding in all four species. The intermediates resemble those of apoMb notably in A and H helices, but differ substantially in the D-, E- and F-helices, which interact with heme. The identified mechanisms cast light on the role of metal/cofactor in poorly understood holoMb unfolding. We also observed β-sheet formation of several myoglobins at 500 K as seen experimentally, occurring after disruption of helices to a partially unfolded, globally disordered state; heme reduced this tendency and sperm-whale did not display any sheet propensity during the simulations.

## Introduction

When proteins misfold or unfold, their functions are impaired or completely lost [1], and specific, condition-dependent intermediates can be formed that reflect the relative stability of subdomains in the proteins [2]. The mechanisms whereby specific proteins unfold remain of central interest, both in order to understand fundamentally the stability and function of natural proteins, to develop recipes for new proteins for industrial or medical use, and to understand neurological diseases related to protein- or peptide misfolding such as Alzheimer's Disease, Parkinson's Disease, and prion diseases [3]–[6].

Myoglobin is one of the most abundant proteins in mammals and of vital importance for O_2_-storage and -transport in muscles [7]–[9]. Holomyoglobin (HoloMb) is ∼77–80% helical as judged from crystal structures, with a typical, mean-residue molar ellipticity [θ]_222_ of ∼−23,000 [10] to −25,000 [11] (units of deg cm^2^ dmol^−1^), due to the eight helices (A–H) constituting its globin fold. At physiological conditions, HoloMb is stable by typically 45–55 kJ/mol (with higher stability observed for Mbs of diving mammals [12], [13]). It unfolds with a barrier of ∼45 kJ/mol (horse Mb) [14] with *T*_m_ ∼80–85°C [11], a process that, in contrast to many other small, single-domain proteins that fold by two-state processes [15], may involve several intermediates (I) between the native (N) and unfolded (U) states [16]. Loss of heme takes days under physiological conditions due to the very high protein-heme affinity [17], with a rate of heme loss of ∼0.01 h^−1^ [18]. The E- and F-helices are probably important for gating the heme group during holoprotein folding/unfolding [19], [20], and the short D-helix seems to contribute to heme retention [21].

In contrast to HoloMb, the unfolding of ApoMb has been studied in great detail as a simple, easily denatured multi-state unfolder. Folded ApoMb contains most of the helices intact with [θ]_222_ ∼−19,000 at neutral pH [22], and −18,000 at pH 6 [23], although the region from the end of the E-helix to the beginning of the G-helix, including the loops on both sides of the F-helix are disordered [24]–[26], consistent with this part of the protein being subject to proteolysis [27]. During acid-induced or denaturant unfolding, mammalian ApoMb, stable by ∼35 kJ/mol [28], generally unfolds via an effectively two-step mechanism [29] with several other, fast steps possible [26] The rate-determining step is the formation of an intermediate I which has lost the C, D, and E helices but retains most of the A, G, and H helices and the C-terminal part of the B helix [30], [31], with [θ]_222_ reduced to −12,000 at pH 4 (where the ellipticity has a shoulder) and down to ∼−5000 at pH 2–3 [22], [23]. 2M Urea reduces [θ]_222_ to −15,000 at neutral pH and quickly denaturates the apoprotein with 3M or at lower concentration in acid [22], [32]

The sizes of the protein states are also very condition-dependent. Their radius of gyration (*R*_g_) increases from 1.8±0.2 nm in HoloMb to 1.9–2.0±0.2 nm in ApoMb and to 2.3±0.2 nm in the ApoMb intermediate (ApoI), as inferred from SAXS [33]–[35], whereas values of *R*_g_ from crystal structures are ∼1.5 nm for both HoloMb and ApoMb. The unfolded state increases in size with lower pH and protein concentration and can be as large as 4.8±0.3 nm when it is a locally extended random coil at pH 1.7 [36].

The N-terminal part of the B-helix and the C- and E-helices fold slowly and are rate-determining for the kinetic stability [37], [38]. To support this, mutations in the E-helix region markedly destabilize N, reduce [θ]_222_, and increase the barrier to folding due to poor packing with the already formed A/B/G/H hydrophobic core [19], but have little effect on I, whereas mutations that impair helix propensity in A (Q8) and G (E109) helices directly reduce the stability of I [32].

Mb was already in the eighties investigated by molecular dynamics (MD), describing the conformational structure and dynamics of the protein [39]–[41]. In the nineties, Mb was simulated at variable conditions, notably temperature [42], explaining the importance of water-protein interactions [43]–[45]. However, very few full-atom folding/unfolding simulations have been carried out for Mb, and only for ApoMb, the first [46] being shortly after the first full-atom protein folding/unfolding simulations at high temperature [47], [48]. These simulations identified loss of D- and F-helices and partial decay of helix ends, and found A, G, and H helices to be most robust in good agreement with NMR and CD data [46], later confirmed by simulations of the individual helices [49]. More recently, simulated unfolding [50] and refolding [51] of ApoMb confirmed the existence of intermediates and characterized these at low pH.

Despite its importance, the mechanism of folding/unfolding of HoloMb has not been studied in detail, except from a very recent study of the electric-field induced unfolding of HoloMb, which produced remarkable stretched intermediates with only 13–20% helicity [52]. The holoMb unfolding mechanism is central to understanding the protein *in vivo* [53] and must differ from that of the apoprotein due to the differences in helix structure and the role of heme. HoloMb has been well-sampled by MD [54], and both the holo- and apo-horse Mb have been investigated in comparison using the same high-resolution structure as the outset, with helix character in good (+/−2%) agreement with experiment [55].

In this work, we investigated the unfolding mechanism of HoloMb to understand how it differs from ApoMb, how heme is detached from the holoprotein, and how this step affects the overall unfolding process. To increase the significance of the simulated behavior, we performed simulations of HoloMb from pig, sperm whale, harbor seal and horse at both 310 K, 400 K and subject to thermal unfolding at 500 K. High temperature favors the entropy associated with heme dissociation and helix unfolding and thus allowed us to investigate these processes in molecular detail. We also performed simulations with both heme fixed to the protein via the Fe-N bond to proximal histidine, and with heme free to move, to evaluate the sensitivity of unfolding to this variation.

The identified mechanism reveals several novel features of holoMb unfolding and heme loss that should be important for understanding *in vivo* life times of the protein, while being also the first comparative study of unfolding/folding mechanisms for orthologous proteins. Notably, in all four simulations, we observed persistence of structural properties consistent with the presence of an intermediate at 2–11 ns, with longer persistence and better preservation of heme and D/E helix structure seen for sperm whale myoglobin. The C- and F-helices were lost quickly in all holoproteins, whereas loss of helices D and E were concerted with heme dissociation. We also interpret the evolution of helix structure in terms of ellipticity and evaluated the effect of helix loss on the deduced ellipticity of the various HoloMb and ApoMb states. Finally, observed substantial β-sheet formation from partially unfolded states of several myoglobin that provide a mechanism for this phenomenon observed experimentally, notably occurring at high temperature from disordered states, with heme preventing sheet formation due to its preservation of helicity.

## Methods

### High Temperature Denaturation

Using high temperature for unfolding simulations has previously been a subject of debate but is now considered a valid approach to unfolding simulations [56]. T should allow a smooth, reversible transition without populating too quickly and indiscriminatingly arbitrary modes [57], and microscopic reversibility is achievable at both high and low simulation temperatures [56]. For a typical barrier of ∼50 kJ/mol, an increase in T from 298 K to 500 K would increase the rate constant by 10^3^–10^4^. More importantly, temperature favors the activation entropy associated with the unfolding transition state: At 500 K, a reduction of the barrier to a few RT will accelerate the unfolding process by ∼10^8^, and the short correlation times will then allow correspondingly shorter simulation times to obtain the same standard errors of observables.

Due to the monotonic scaling nature of T, high- and low temperature is theoretically expected [58] and computationally confirmed to give similar folding/unfolding pathways [59]–[61], as also seen experimentally [62]. The often cited example to the contrary by Karplus et al. [63] did not show that high and low T gives different pathways, which would conflict with the these results, but instead showed that for barnase, and probably for other proteins, acid and thermal denaturation give different pathways, most likely because the first requires molecular modification (uptake of protons at specific sites), whereas the other is a uniform scaling of all interactions. HoloMb takes up ∼6 protons during acid unfolding [10]. Several titrated histidines, possibly His-24, His-64, and His-113, are thought to contribute ApoMb unfolding in acid [28], [64]. A common reason for not performing heat denaturation to experimentally study unfolding is the aggregation tendency at higher T [2], which is not an issue in simulations.

HoloMb is substantially (>10 kJ/mol) more stable than ApoMb, so while simulation at 350–400 K may be adequate for ApoMb unfolding, much higher simulation temperature is needed to achieve heme loss and holoMb unfolding. To model heme loss at 500 K, we both considered heme fixed and free in different simulations. We probed the distance of Fe atom of the heme group to the center of mass of His93 residue. At 300 K, this distance is on average ∼0.64 nm for equilibrated structures. We then used a two-times larger value of 1.28 nm as the threshold for heme loss in simulations. Using this criterion, in the two simulations done for each protein at 500 K, pig, horse, seal and sperm-whale Mbs loss their heme after ∼1.0 and 1.8 ns, 1.0 and 1.6 ns, 1.7 and 3.0 ns, and 3.7 ns, respectively (Table S9 in File S1). We performed additional simulations at both 400 K and 500 K to confirm the observation that heme was more retained in sperm whale myoglobin.

### Simulation Protocols

MD unfolding simulations were performed for Mbs of sperm whale, pig, horse, and harbor seal, using the GROMACS package [65] and the starting crystal structures 1U7S [66] (resolution 1.4 Å), 1PMB [67] (resolution 2.5 Å), 1YMB [68] (resolution 1.9 Å), and 1MBS [69] (resolution 2.5 Å). It is notable that previous apoMb studies have used holoMb starting structures which have much higher helicities. Knowing that the apoprotein is highly disordered in some regions, e.g. the F-helix, this may impact the realism of such simulations, but this is no issue in this work, which constitutes the first holoMb unfolding simulation based on holoMb crystal structures.

After careful consideration, we decided to describe all the proteins by the GROMOS 96 (43a1) force field [70], which has been shown by two independent research groups to give a good balance of helicity and sheet propensity in comparison to other commonly used force fields [71], [72], of particular relevance to this study. The protein was solvated with water described by the SPC explicit solvent model [73]. Side-chain ionization states for all cases were adjusted to a pH of 7.0 using the Propka web server [74]. The proteins were placed in cubic unit cells with the size of 6.7×6.7×6.7 nm^3^, large enough to contain the macromolecule and ∼1.0 nm of solvent on all sides. To obtain a neutral simulation system, Na^+^ or Cl^−^ ions were added by randomly replacing the water in the simulation cell. Standard periodic boundary conditions were applied with particle-mesh Ewald electrostatics [75] for the calculation of non-bonded interactions. The short-range cutoff radius was set to 1.0 nm and the cutoff radius for both Coulombic and Lennard-Jones interactions was 1.4 nm.

The system was energy minimized with a steepest descent method for 400 steps and a 10 kJ mol^−1^ nm^−1^ energy tolerance for the convergence of the minimization process. This was used as the initial configuration of a 500 ps position-restraint simulation in which the protein was harmonically restrained with an isotropic force constant of 1000 kJ mol^−1^ nm^−2^. This allows further equilibration of the proteins and solvent while keeping the conformation of the protein unchanged. To simulate the system in an NPT ensemble, a fixed-volume simulation cell was applied with a Nosé–Hoover thermostat [76], [77] having a relaxation time of 0.1 ps. The protein position-restraint was then removed and MD simulations were performed for 10 ns at a temperature of 300 K and a pressure of 1 bar using the leap-frog algorithm to numerically integrate the equations of motion with a time step of 2 fs. LINCS [78] and SETTLE [79] algorithms were used to constrain the bond lengths in non-water molecules and water molecules, respectively. Interactions within the short-range cutoff were updated every time step whereas interactions within the long-range cutoff were updated every ten time steps using a neighbor list and gridding of the simulation cell. All atoms were given an initial velocity obtained from a Maxwell distribution at the desired initial temperature.

To simulate the high-temperature unfolding of the proteins, all structures were also subject to simulations at both 400 K and 500 K, and an additional seeded simulation was done at both temperatures to confirm the general nature of heme loss and helix denucleation.

To put the results into experimental context, helix character was converted into mean molar ellipticity and monitored as described below (the sensitivities of parameters are described in Figures S1 and S2 in File S1). Also, the secondary structure types were plotted for all four proteins at 310 K (Figure S3 in File S1), to confirm the integrity of all eight helices in the simulated HoloMb structures. Structural overlaps and contact maps compared for experimental crystal structures used for each protein and equilibrated proteins are given in Figure S4 in File S1. Secondary structure plots for additional simulations at 400 K and 500 K are presented in Figures S5–S19 in File S1). Simulations were performed both with heme fixed to proximal histidine and without restraint, to test sensitivity to this parameter. Secondary structure assignment was performed with DSSP [80]. Within the DSSP framework, intra-backbone hydrogen bonds are identified based on pure electrostatic interactions, and secondary structure is assigned from the repetition patterns of hydrogen bonds.

### Computing the relationship between protein structure and ellipticity

The mean residue ellipticity at 222 nm, [θ]_222_ is mainly due to the n-π* transitions of helices [11], [81] and can thus be calculated from the assigned helix character of the residues in the protein [82], with four parameters that are all approximately experimentally known for the holoMb native state (units of deg cm^2^ dmol^−1^):
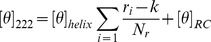
(1)
*N*_r_ is the number of residues in the protein (153), *N*_h_ is the number of helices (8 in HoloMb) and *r*_i_ is the number of residues in the helical conformation for each helix *i*, calculated using the DSSP algorithm [80]. [θ]_helix_ is the signal at 222 nm for a complete, infinite helix, and when divided by *N*_r_, it gives the average signal per residue for an infinite helix. We compared both the value −39,500 optimized for the infinite helix and the value −36,800 optimized for Mb [82]. The empirical parameter *k* is a correction term for helix truncation that reduces the CD signal, with typical values from 2 to 5 [83], [84]. A value of 3 was used in previous works [85], . Here we use both *k* = 3 and *k* = 2.57 as an optimized value [82].

We tested the sensitivity of all four parameters to the calculated relationship between [θ]_222_ and Mb structure (See Figures S1 and S2 in File S1). We also corrected the predicted [θ]_222_ for the absorption of random coil at 222 nm, [θ]_RC_, estimated at +1000 deg cm^2^ dmol^−1^ for the folded state and increasing proportionally with its fraction (1−*f*_H_). Sensitivity to this correction is minor, as seen in Figure S2 in File S1. Importantly, as shown below, the fraction of helix character *f*_H_ is not a simple linear function of [θ]_222_ as generally assumed, since some helices are completely absent in the ApoMb and in intermediates, compared to the HoloMb (i.e. proper values for the number of helices should be used).

### Clustering analysis

We used k-means clustering as implemented in MATLAB, vR2010a, which partitions data into *k* mutually exclusive clusters and returns the index of the cluster. RMSD and number of main-chain-main-chain hydrogen bonds were used as reaction coordinates representing changes in tertiary and secondary structures, respectively. The distance of all members to their respective centriod is minimized and used to define each cluster iteratively. The objects are further allowed to move between different clusters until the distance metric cannot be reduced further.

## Results and Discussion

### Reconciliation of experimental data for myoglobin states

To facilitate the discussion of our simulation results, we compiled available experimental data for Mb states from circular dichroism, crystal structures, and small-angle X-ray scattering (SAXS) in Table 1. Calculated SASA and radius of gyration (*R*_g_) from crystal structures were based on averages of the structures 1A6M [87], 1MBO [88], 1U7S [66], and 1U7R [66] for HoloMb. Notice that *R*_g_ values from SAXS are ∼0.3–0.4 nm larger than those from crystal structure, with the truth probably being somewhere in between (crystal packing forces may reduce *R*_g_ in crystals). Despite these uncertainties it is clear that the ApoMb is not significantly expanded compared to HoloMb, despite its considerable loss of helix character: The fraction of helix character *f*_H_ of HoloMb structures is generally seen to be ∼0.77–0.80 (see further discussion below), whereas that of ApoMb was estimated to be 0.53 [89].

**Table 1:** Experimental Data for Myoglobin States from the Literature.

State	Conditions	[θ]_222_ (deg cm^2^ dM^−1^)	f_H_ (%)	SASA (nm^2^)	R_g_ (nm)
HoloN	pH 7	−23,000 [9]; −25,000 [10]	78^a^	80.26^a^	1.52^a^ (X-RAY); 1.8±0.2 [32], [33] (SAXS)
ApoN	pH 7	−19,000±1000 [21]	53 [86]	—	1.9±0.2 [34] (SAXS)
ApoI	pH 4, 0M Urea	−12,000±1000 [21]	<0.41^b^	—	2.3±0.2 [34] (SAXS)
ApoU	pH 5, 4.5M Urea	−3000±1000 [21]	∼0	—	3.4±0.2 [34] (SAXS)

^a^ For HoloMb, average based on crystal structures 1A6M [84], 1MBO [85], 1U7S [65], and 1U7R [65].

^b^ Estimated from NMR data, see text.

Estimates of [θ]_222_ of HoloMb vary by ∼2000, giving an idea of the uncertainty in this observable (this uncertainty is also consistent with our thermal fluctuations in [θ]_222_ of ∼1,500). As ellipticity changes with increasing denaturant or lower pH, there is also evidence of some expansion of ApoMb. The properties that correspond to the intermediate ApoI, which has been characterized extensively as described in the introduction, are likely to be those representing a shoulder in the ellipticity at −12,000 for the “acid intermediate” at pH 4 [22], [23]. NMR-data suggest that most of the A, G, H, and part of B-helices are intact in ApoI [30], [31]. Using 16, 19, and 26 residues in A, G, and H-helices and two residues remaining in the B-helix, *f*_H_ is ∼0.41 for ApoI, as we have noted in Table 1. This value of *f*_H_ is fully consistent with an ellipticity of −12,000 estimated from CD [22], based on our calculations with *k* = 2.57, *N*_h_ = 4, and [θ]_helix_ = −36,800, providing excellent consistency between CD, X-ray crystal, and NMR data for both HoloMb, ApoMb and ApoI.

Table 2 gives an overview of our estimates of *f*_H_ from experimentally determined [θ]_222_. We used a correction for [θ]_222_ by the random coil fraction 1−*f*_H_ of +1000. Although not considered before, this fraction increases for more unfolded states as *f*_H_ decreases but by a modest ∼0.01 compared to a value of zero. The sensitivity to the choice of the helix-truncation correction factor *k* is maximal for the fully folded HoloMb where *f*_H_ is largest and amounts to 0.02 when changing *k* from 2.57 to 3.00.

**Table 2:** Calculated helix fractions f_H_ from ellipticities of myoglobin states when accounting for decreasing N_h_ for two values of the infinite helix (one general and one for Mb) (N_r_ = 153, k = 2.57, [θ]_222_ for random coils = +1000).

	Holo, pH 7	Apo, pH 7	Apo, pH 6	ApoI, pH 4	I/U
Experimental [θ]_222_	−24000	−19000	−17500	−12000	−5000
N_h_ (# helices)	8	6	5	4	2
f_H_ with [θ]_helix_ = −39500	0.75	0.59	0.54	0.39	0.18
f_H_ with [θ]_helix_ = −36800	0.79	0.63	0.57	0.41	0.19
# helix residues	122	97	88	63	27

Two effects are however very important for determining *f*_H_ from experimental ellipticities: The value of [θ]_222_ for the infinite helix, [θ]_helix_, and the actual number of helices *i* in each Mb state. Changing [θ]_helix_ from −39,500 to −36,800 (the first being estimated for a general helix, the deduced for Mb data [82]) changes *f*_H_ by up to 0.05 or eight residues (most for states with high *f*_H_ when correction for random-coil absorption is small). Thus, *f*_H_ computed for both values of [θ]_helix_ are given in Table 2. However, it is notable that the Mb-optimal parameter choice [θ]_helix_ of −36,800 recovers the *f*_H_ of HoloMb of ∼0.78 expected from crystal structures (Table 2), so we will discuss *f*_H_ calculated from this value in the remainder of the paper.

Previously, the number of helices has, like the increasing random-coil absorption, not been considered in estimates of *f*_H_ for myoglobin states. This correction can also be substantial: For example, an ApoI state with [θ]_222_ = −12,000 would have *f*_H_ changing from 0.45–0.47 when using *i* = 8 to the more correct 0.39–0.41 when using *i* = 4 (as used in Table 2), a difference of 0.06, or a 15% overestimation of helicity for ApoI if not correcting for the lower helix number. Similarly, ApoMb would reasonably have *i* = 6 (considering the loss of helicity in part of the E-F-G region), which reduces *f*_H_ estimated for ApoMb by 0.03, amounting to 4–5 less helical residues. Our best estimate is thus that ApoMb in a state with [θ]_222_ = −19,000 will have *f*_H_ = 0.63, corresponding to a loss of 23–26 helical residues and effectively two helices. An intermediate with [θ]_222_ = −12,000 will have *f*_H_ = 0.41, with helical residues reduced from 120–122 in eight helices for HoloMb to 62–64 helices in four helices (A, G, H, and part of B). Correspondingly, a value of −5000 as seen in some experimental denaturation studies could then realistically reflect *f*_H_ = 0.19 or 0.21 with a total of 28–33 residues left in 2–3 helices. With these calculations, we now turn to the discussion of our simulation results.

### General properties of simulated myoglobins

**Figure 1:**
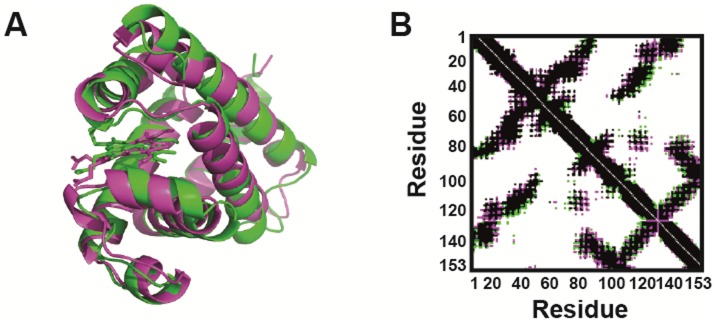
Experimental vs. simulated structure at 300 K. A) Structural overlay of the MD-averaged structure (pink) and the crystal structure of sperm whale Mb (PDB: 1U7S [66], green). B) Contact maps of the MD-averaged structure and the crystal structure. Contacts unique to MD and crystal structures are shown in green and pink, respectively. Shared contacts are marked in black, with 91.9% similarity between the structures.

Figure 1A shows the MD-averaged structure of sperm whale Mb (pink) overlayed with the crystal structure of sperm whale Mb (PDB: 1U7S [66], in green), and Figure 1B shows the corresponding contact maps of the MD-averaged and crystal structures (further averaged properties such as SASA and hydrogen bond counts are given in Tables S1–S8 in File S1). The contacts that are unique to the MD averaged structure and the crystal structures are shown in green and pink respectively contacts. Black contacts show the shared contacts. Overall, the simulated and crystal structures are in very good agreement, showing that the MD-averaged 310 K structures are robust reference points for the thermal denaturation that we will discuss in the following.

All four orthologs were also simulated at 400 K, to see the temperature-effect of the general structure and dynamics of the proteins. Secondary structure-evolution of these simulations together with those at 500 K are given in Figures S5–S19 in File S1. At 400 K, most of the secondary structure remains intact and heme is not lost, neither in simulations where heme is fixed via iron to the proximal histidine, nor in those where it is not, the reason being that the entropy of heme release is not yet large enough to initiate holoprotein unfolding.

At 400 K, whereas sperm whale (Figures S7 and S8 in File S1) and harbor seal (Figures S18 and S19 in File S1) myoglobins were more robust regardless of heme fixation, those of in particular pig (helices A, B, E, and F, Figure S11 in File S1) and to some extent horse (residues 40–60, Figure S15 in File S1) showed loss of secondary structure. Still, the D and E helices remained intact due to their association with heme, which is in understandable contrast to the apoprotein where these helices are less stable. The results at 400 K could suggest more stable helices in the proteins of the aquatic mammals, although the 500 K simulations (*vide infra*) with the full unfolding process will describe these trends more fully.

**Figure 2:**
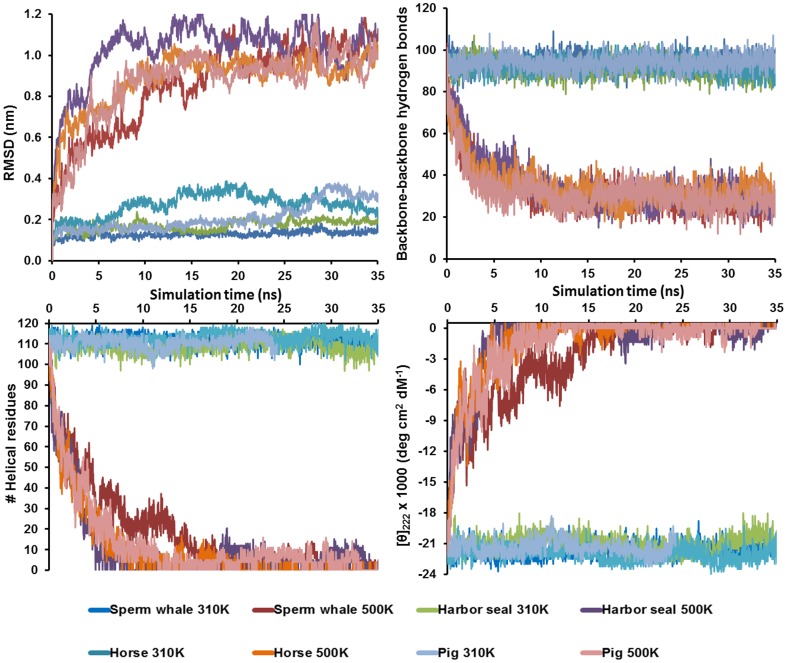
Properties of myoglobins studied in this work during the first 35 ns at 310 K and 500 K. A) RMSD for C^α^-atoms vs. crystal start structure; B) backbone-backbone hydrogen bonds; C) Number of helical residues; D) Computed Molar mean-residue ellipticity at λ = 222 nm.

Figure 2 shows the time-evolution of several properties for the 310-K simulations (in bluish colors) and the 500-K simulations (in reddish colors). The root-mean-square deviations (RMSD) of the C^α^-atoms with respect to the initial crystal structure at 310 K are given to the left in Figure 2A. At 310 K, all four proteins were stable and folded along the trajectories. Sperm whale (dark-blue) HoloMb remained close to the starting conformational structures thoughout the simulation (RMSD ∼0.12 nm). Harbor seal (green) remained within RMSD<0.2 nm but displayed larger some conformational changes along the simulation, representing dynamics relative to the fixed crystal structure. Pig and horse (light-blue and cyan) HoloMbs displayed substantial dynamic changes in conformational structure with RMSD values reaching up to 0.36 nm. The conformational changes anti-correlated with the resolution of the starting crystal structures: The sperm-whale HoloMb crystal structure has a near-atomic resolution of 1.4 Å [66] giving very stable conformational dynamics, whereas the other structures with subatomic resolutions 1.9–2.5 Å gave rise to substantial reorientation of the helices and loops, as reflected in the RMSD curves, Figure 1A.

The time evolutions of the backbone-backbone hydrogen bonds, reflecting the helix structure, are shown in Figure 2B, and the number of residues assigned to be helical is shown in Figure 2C. For all these three structural properties we infer that at 310 K, the proteins remained near the native state during the simulations, with ∼105–120 helical residues (*f*_H_ ∼0.69–0.78), slightly less than in crystal structures due to the dynamic loss of helix ends in aqueous solution.

Correspondingly, the radius of gyration (*R*_g_) was ∼1.51 nm for sperm whale and seal Mb and ∼1.48 and 1.50 nm for pig and horse Mb, averaged from 30–35 ns, in good agreement with the value of 1.52 nm from the average of *R*_g_ for crystal structures of sperm whale and seal Mbs (PDB IDs: 1A6M [87], 1MBO [88], 1U7S [66], 1U7R [66], and 1MBS [90]).

Figure 2D shows the time-evolution of [θ]_222_ computed from Equation (1), corrected for helix truncation, using [θ]_helix_ = 36,800. These data provide an account of the change in expected [θ]_222_ upon unfolding of HoloMb, with an average value of ∼−22,000 at 310 K, similar for all four proteins and slightly less than the experimental estimate, as also deduced from the helical character above. We also obtain estimates of the dynamic nature of [θ]_222_, which is subject to thermal fluctuations of approximately ±1500 during the simulations.

### Thermal unfolding of holomyoglobins

The four simulations at 500 K led to gradual disruption of helices to form fully disordered structures within the first 20 ns. Figure 2 shows the time-evolution of the properties of these unfolding proteins in red (sperm whale), orange (horse), pink (pig), and violet (harbor seal). As shown in Figure 2A, during the simulations at 500 K, the RMSD of C^α^-atoms increased to ∼1 nm and stabilized at this level. During these conformational changes, the number of backbone-backbone hydrogen bonds decreased from ∼90 to ∼30 more or less uniformly for all proteins (more data are given in Tables S1–S8 in File S1). Consistent with this, the computed [θ]_222_ decreased as secondary structure was lost. Due to the limited accuracy in the unfolded limit, viz. the behavior of Equation (1) as *N*_h_ goes to zero, we truncated the measured [θ]_222_ at +500, but all trajectories approach the limit of the random coil absorption specific to the choice of this parameter, in the range 0–5000 (Figure S2 in File S1). It can be seen that overall, θ_222_ decreases exponentially in a way that is similar for all four proteins and suggest an exponential decay of the folded state, i.e. the unfolding process can be considered a first-order process.

However, as a deviation from exponential decay, the RMSD curves, the ellipticity, and the number of hydrogen bonds displayed plateaus for all four proteins between 2–10 ns. Since we observe this plateau in all four proteins for three different properties, we consider this observation significant. We interpret it as indicative of cooperativity deviating from exponential decay, due to an intermediate which is kinetically robust so that structure is not degraded exponentially along the reaction coordinate. In contrast, a smooth transition to the unfolded state with no persistence of properties along the trajectory would indicate a process with no well-defined intermediate.

From the RMSD curves at 500 K (Figure 2A), the plateaus can be seen to be longest in time for sperm whale Mb, spanning from 5–11 ns, and shortest (2–3 ns) for pig and horse Mb. Also, the plateau of the sperm whale Mb has the lowest RMSD relative to its 310-K native structure, which indicates a native-like conformation in this range of the simulations when heme remains bound to the proximal histidine. In Figure 2B, a plateau in the persistence of backbone-backbone hydrogen bonds suggests that secondary structure is roughly unchanging from 3–7 ns. This is confirmed by the assigned helix character of the proteins (Figure 2C). From the ellipticities in Figure 2D, to be discussed in more quantitative detail below, retention is seen in the 2–7 ns range for sperm whale myoglobin where heme is bound to proximal histidine and in the 2–4 ns range for the other proteins. This information is derived from the persistence of helical residues and confirms that helix character is approximately exponentially decaying, with sperm whale Mb having about 10 more helical residues in the intermediate range from 5–11 ns, where a plateau or even reformation of helix character is evident, directly showing the importance of the interplay between heme retention and helix robustness.

**Figure 3:**
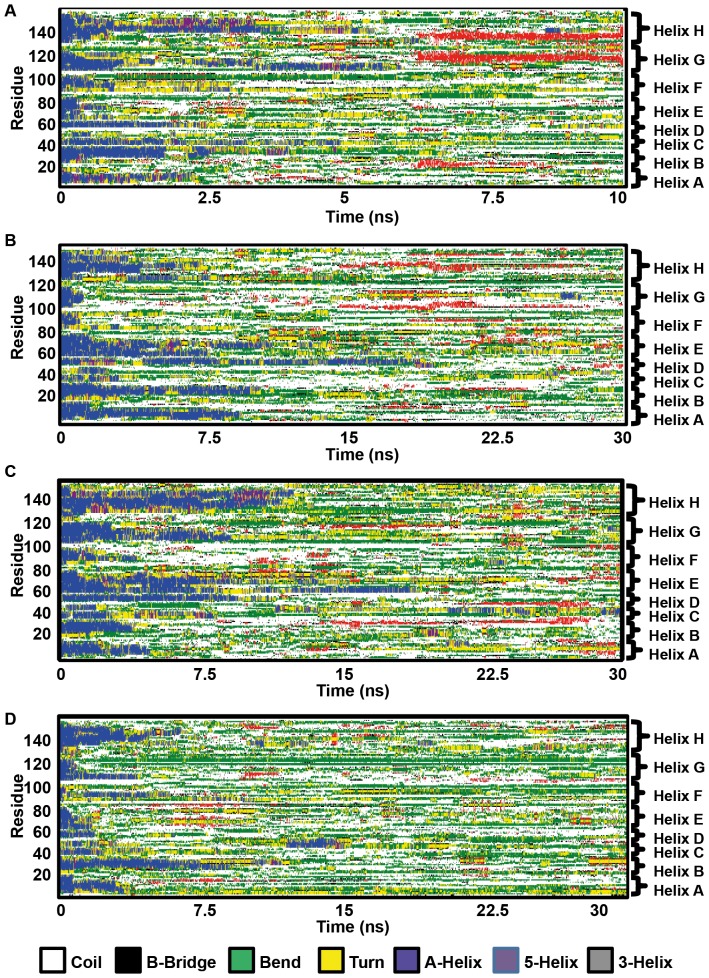
Secondary structure changes during thermal unfolding for holomyoglobins. A) harbor seal; B) sperm whale; C) pig; D) horse. The approximate location of helices is shown to right. Plots were made with the DSSP program implemented in Gromacs.

To understand better the secondary-structure loss during thermal unfolding, we computed the structural character of the residues as a function of simulation time at 500 K, as shown in Figure 3: harbor seal Mb (Figure 3A), sperm whale Mb (Figure 3B), pig Mb (Figure 3C), and horse Mb (Figure 3D). All eight helices are clearly visible in the beginning of the simulations, marked as blue color to the left of Figure 3. Helix loss begins immediately upon heat denaturation and continues until 10–15 ns. The D/E-helix segment tends to persist long in HoloMbs, due to its association with heme (*vide infra*), in contrast to ApoMb. In some cases there are sporadic and non-significant recurrences up to 20 ns. The helices become largely bend (green), coil (white), and turn (yellow) along the unfolding simulations. However, there are also many β-bridges (single-pair interactions) and sheets (at least two pairs) forming during the course of the simulations, to be discussed below.

The only helix that is fully lost within 5 ns in all four proteins is the F-helix, consistent with it being disordered in ApoMb [24], [25] and with previous simulations indicating this helix to be unstable [49]. Furthermore, the C-helix is quickly lost in all simulations, consistent with the higher stability of other helices (notably A/G/H that are preserved in ApoI) [91], [92]. In addition to the D-helix, both A- and H-helices exhibit better preservation, a feature also seen for ApoMb, which is meaningful as these residues interact relatively little with heme and should thus be robust in both holo- and apoMb. Sperm whale Mb (Figure 3B) presents the best preservation of the A and B helices (9 ns and 10 ns, Figure 2B), which are lost within 3–8 ns in the other proteins. Sperm whale and pig Mbs preserve the D- and E-helices best, and pig Mb preserves the G and H helices substantially better (up to 10 ns) than the other proteins. Overall, horse Mb displays the fastest loss of helices, and pig and sperm whale Mbs display the highest persistence, however in different ways: Pig Mb preserves the G/H/E helix whereas sperm whale Mb preserves A/B/D/E. Taken together with the simulations at 400 K, sperm whale holoMb thus displays comparatively high preservation of structure.

The persistence of helices A, B, and D are responsible for the longer preservation of total secondary structure and the more persistent shoulder in the computed ellipticity of sperm whale Mb. The heme-interacting D- and E-helices are best preserved by sperm whale Mb where heme remains bound to proximal histidine, which directly shows the importance of heme retention for helix preservation. Indeed, these two helices, together with the loose, gating F-helix, are also known from mutation studies to be important for heme retention in HoloMb [19]–[21].

Overall, the simulations then display a common trend of early loss of F- and C-helices, and heme dissociation occurring in concert with D- and E-helix loss, which then leads to full denaturation. This resembles the ApoMb unfolding pathway, where ApoI has A/G/H/B character, except for the additional stability of the D- and E-helices in particular for sperm whale and pig Mb, which interact with heme to keep this part more stable than in ApoMb. The second exception is helix A, which seems to depend on the integrity of the heme/D/E nucleus, being most robust in the structures where these are intact (Figure 3B and 3C).

**Figure 4:**
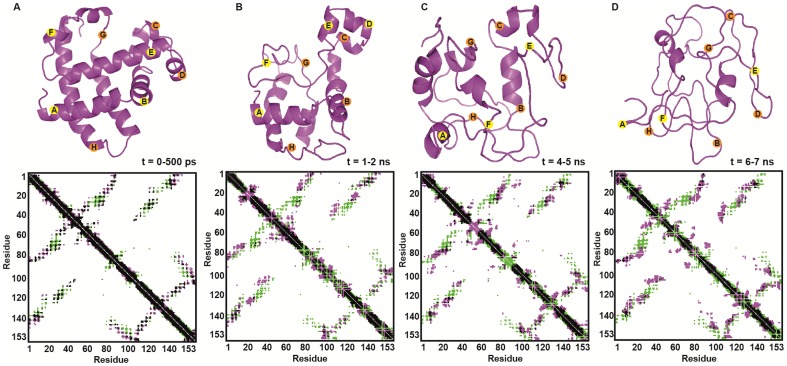
Snapshots (top) and contact maps (bottom) of average MD structures at 500 K. A) t = 0–500 ps, B) t = 1–2 ns, C) t = 4–5 ns, and for D) t = 6–7 ns. Contacts unique to the crystal structure are shown in green and contacts unique to the protein at different simulation times are shown in pink. Beginnings of all helices are marked with yellow alphabets (helices in front) and orange alphabets (helices in back).

The course of the thermal unfolding is summarized using contact maps computed from the average sperm whale Mb structures in Figure 4. The rate-determining N→I step in ApoMb unfolding most likely involves partial loss of helices B and loss of helices C, D, and E [26] (the two-dimensional RMSD matrix for this simulation is given in Figure S20 in File S1). Comparing our computed holoMb unfolding processes to the in principle six possible states discussed by Olson and co-workers [16], show that of the six possible states (ApoN, ApoI, ApoU, HoloN, HoloI, HoloU), the simulated HoloMbs never enters the ApoMb pathway except in the fully unfolded state, i.e. heme loss is coupled to helix loss, as explained above, and HoloI has substantially more D- and E helix structure intact (see secondary structure plots in Supporting Information).

**Figure 5:**
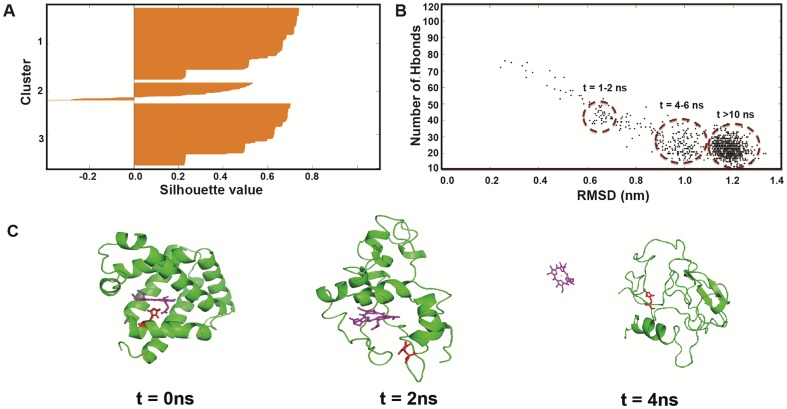
Clustering analysis of distinct intermediates. A) Silhouette graph for sperm-whale myoglobin at 500 K using K-mean clustering confirms the existence of three distinct intermediates when using main-chain-main-chain hydrogen bonds as descriptor; B) 2D plot of the number of main-chain main-chain hydrogen bonds vs. overall RMSD, revealing the three distinct clusters of structural forms; C) Corresponding heme loss during thermal unfolding of sperm whale holoMb at 500 K.

As a further analysis of the probable intermediates, we considered the clustering of structural forms in all four myoglobins with freely dissociating heme as described in the Methods section using k-means. Three clusters could be defined as shown for sperm whale myoglobin in Figures 5A and 5B, using the number of main-chain main-chain hydrogen bonds, describing secondary-structure loss, and the overall RMSD. Very similar clusters, although at somewhat different timings, were seen for the other proteins (Figures S21–S24 in File S1), although harbor seal holoMb only resulted in the two latest, significant clusters. The similarity of the clusters suggest a common general pathway of unfolding.

To check whether the defined clusters were separated or not, a silhouette plot was constructed (Figure 5A), which displays how close each point in the cluster is to points in neighboring clusters. The measure ranges from +1, indicating points that are very distant from neighboring clusters, through 0, indicating points that are not distinctly in one cluster or another, to −1, indicating points that are probably assigned to the wrong cluster. The silhouette graph confirms the existence of three clusters, with the first cluster as the most distinctive one. The cluster centers were located at (RMSD = 1.15, 27 hydrogen bonds remaining), (RMSD = 1.17, 20 hydrogen bonds remaining), and (RMSD = 0.67, 41 hydrogen bonds remaining), respectively. The clusters occurred roughly at 1–2 ns, 4–6 ns, and after 10 ns (Figure 5B). Figure 5C shows how these clusters relate to heme loss along the unfolding coordinate, showing that heme dissociation occurs in relation to these changes in secondary structure to form unique HoloMb intermediates of partial heme and helix loss.

### Implications of HoloMb unfolding for *in vivo* kinetic stability

Early work tried to rationalize the high stability of diving mammal Mbs by comparing sequence differences, showing that electrostatic energies could not explain the extreme stability of sperm whale Mb, whereas hydrophobic interactions were suggested to be important for stability [12], [93]. In previous work using an improved protocol [94], [95] of the stability estimator FoldX [96] to calculate Mb stabilities evolved for cetaceans and terrestrials, four sites (5, 35, 66, and 121) were under positive selection and could explain a significant part of the evolved stability of the cetaceans [97]. 10–20 fold increases in total [Mb] is a known adaptation of cetaceans to increase dive length upon specialization within the aquatic niche. Selection for ∼10 kJ/mol higher stability, known experimentally [38], was then explained as due to selection against the otherwise also 10–20 fold larger copy-number of unfolded proteins, since higher stability reduces the burden of misfolded Mb to the same level as in terrestrial cells [97].

In our simulations, we identified a coupling between heme retention and helix preservation, as particularly evident in sperm whale Mb (Figure S5 vs. Figure S6 in File S1) and pig Mb (Figure S9 vs. Figure S10 in File S1). This constraint increased the robustness of helix B, D and E. Also, HoloMb differs from APoMb in its unfolding process due to heme loss being coupled to helix loss, a coupling largely governed by D- and E-helices, suggesting that the higher stability of sperm whale Mb could be partly due to stronger persistence of interactions between heme, D- and E-helices. Even for ApoMbs, the D- and E-helices should be crucial as they are lost in the rate-determining unfolding step [26], and in HoloMb, their interaction with heme is the major determinant of kinetic stability against thermal unfolding. It was previously found [98] that HoloMetMb folding and unfolding rates could be described for two pathways depending on heme orientation. It was also found that even in the unfolded state, heme remains coordinated, which is consistent with its high affinity for the apoprotein [98] and consistent with the our simulations, where heme always remain bound to some parts of the proteins, although no longer in the native conformation.

### β-sheet formation in myoglobins at high temperature

A last observation from our simulations that deserve mentioning was the finding of β-sheet formation in some proteins, notably seen beginning at ∼5 ns in pig Mb (Figure S10 in File S1) and in harbor seal Mb (Figure 3A and Figure S17 in File S1). Sperm whale Mb showed the smallest tendency to form sheets across all simulations (Figures S5/S6 in File S1). The sheets were formed mainly from the residues that previously made up helices A, G and H (Figure 3A for harbor seal Mb; Figure S10 in File S1 for pig), and to some extent E helices (e.g. Figure S17 in File S1), which are also among the most robust helices in myoglobins. The sheets tended to form more when heme was dissociated from the protein (Figure S10 vs. Figure S9, Figure S12 vs. Figure S13, and Figure S16 vs. Figure S17 in File S1), suggesting its role in preserving the local helical structure interact. The process required the helices to be lost and change to a disordered form before the sheets could be formed. While different force fields have different sheet and helix propensities, Gromos has been shown to perform well in its respective benchmarks on helix/sheet propensity balance [71], [72], making the observation of sheet formation from partially disordered states of a helix protein, as seen here, more relevant.

β-sheet formation, in particular in the form of amyloids, is a hallmark of many neurodegenerative diseases and such structures form under conditions where the native state is unfavored [3], [4], [99] either due to genetic mutations (“heritage” component of disease) or due to stress-induced post-translational modifications (“environment” component of disease), sometimes playing together to increase the burden of misfolded peptide or protein [3], [99]. It has been found that ApoMb under thermal denaturation can form amyloid fibrils by interaction of disordered parts of an unfolded ensemble that lack strong secondary structure hydrogen bonds and are thus susceptible to β-sheet formation [100]. In this context, we decided to also report this observation, which seems to suggest a mechanism whereby stable helices are disrupted to a certain point where they then start to form sheets (it is notable that many amino acids, incidentally, have high propensities for both sheet and helix formation).

### Conclusions

In conclusion, we have performed more than twenty 20–30 ns simulations of holoMb from four different species, two aquatic and two terrestrial, which constitute the first systematic study of HoloMb unfolding and is also the first comparative unfolding/folding study of protein orthologs from different species (sperm whale, pig, horse, and harbor seal). Simulations were done at 310 K, 400 K, and 500 K, and with heme either bound to the protein or free. Agreement with experimental structural data (alignment, contact maps, and helix integrity) for each separate protein was first confirmed at 310 K (Figure 1, and Figures S3–S4 in File S1), and simulation results were interpreted in relation to experimentally ellipticies from CD (Figure 2D).

At 400 K, most secondary structure remained intact and heme was not dissociated from the protein regardless of whether the cofactor was fixed to proximal histidine or free to move, i.e. at 400 K, the entropy of heme release was not yet large enough to initiate holoprotein unfolding. Sperm whale (Figures S7 and S8 in File S1) and harbor seal (Figures S18 and S19 in File S1) myoglobins were more robust regardless of heme fixation, whereas pig (Figure S11 in File S1) and to some extent horse (Figure S14 in File S1) lost some secondary structure already at 400 K, but the D- and E-helices remained intact and associated with heme. This could suggest more stable helices in the proteins of the diving mammals, as recently suggested due to prevention of myoglobin misfolding [97].

At 500 K, the C- and F-helices were lost quickly in all cases. The protein conformations (measured by the RMSD relative to the starting structures), the secondary structure (number of helical residues and number of backbone-backbone hydrogen bonds), and the molar residual ellipticity decayed exponentially and were all found to display plateaus between 2–11 ns. We interpreted these plateaus as intermediates of HoloMb unfolding common to all four species, although with substantial differences in the details, as shown in Figure 3. Three distinct structural forms (albeit only two for harbor seal Mb) corresponding to before, during, and after heme loss were confirmed by k-means clustering analysis, and supporting a mechanism of heme-release coupled secondary structure decay. The intermediates in several ways resemble those of ApoMb, notably in G and H helices, but differ substantially in the D-, E- and F-helices, which interact with heme (the F-helix is already disordered in ApoMb, partly explaining this difference). To further understand this, we simulated all proteins both with heme free or fixed to proximal histidine. The fixation of heme delays loss of C, D, E, and H helices in sperm whale Mb (Figure S5 vs. Figure S6 in File S1) and this protein overall displayed the strongest persistence of secondary structure in D- and E-helices, showing the role of heme in preserving overall secondary structure during unfolding.

Small to moderate β-sheet formation as measured by the DSSP assignment was seen in all proteins at 500 K. Sperm whale myoglobin was remarkably resistant to sheet formation in most simulations. Sheet was most dominant when heme was freely lost, implying that heme association with the D- and E-helices reduce sheet formation, consistent with a mechanism where β-sheets are formed from globally partially disordered protein states after disruption of all helices.

In summary, the identified mechanisms cast light on the role of the metal/cofactor during holoprotein unfolding and show that the metal cofactor release is coupled to holoprotein unfolding, preserving D- and E-helices and preventing β-sheet formation from partially unfolded, globally disordered states, explaining why the holoproteins are more robust to unfolding than the apoproteins. The simulations, by comparing four orthologs, reduce the role of systematic errors commonly found in MD-simulations. They suggest that sperm whale myoglobin is more robust to β-sheet formation and has more robust helices also at 400 K, when compared to proteins from horse and pig.

## Supporting Information

File S1 **File S1 contains information about the relation between helix character and ellipticity (Figure S1); figures showing the sensitivity to variation in parameters (Figure S2); Secondary Structure types at 310 K for all four proteins (Figure S3); correlation of structures to experimental structures (Figure S4); Time evolution of secondary structures (Figures S5–S19); 2D RMSD matrix for alpha carbon (Figure S20); Key average properties of the simulated proteins separated into helices (Tables S1–S8); Timings of heme loss from simulations at 500 K, first and second replicate (Table S9); clustering analysis for all four proteins (Figures S21–S24).**
(PDF)
