# Supplementary material for: Unfolding Simulations of Holomyoglobin from Four Mammals: Identification of Intermediates and β-Sheet Formation from Partially Unfolded States
Source: PLoS One. 2013 Dec 27;8(12):e80308. doi: 10.1371/journal.pone.0080308 (PMC3873898; doi:10.1371/journal.pone.0080308)

SUPPORTING INFORMATION

Unfolding simulations of holomyoglobin from four mammals: Identification of intermediates and β-sheet formation from partially unfolded states

Pouria Dasmeh[a,b] and Kasper P. Kepp[a]*

[a] *Department of Chemistry, Technical University of Denmark, Kongens Lyngby, Denmark*

[b] *Max Planck Institute of Immunobiology and Epigenetics, Freiburg, Germany*

* Corresponding author. E-mail: kpj@kemi.dtu.dk. Phone: +045 45 25 24 09.

Figure S1. Relation between ellipticity and fraction of helix f_H, for myoglobins with helix number i = 1−8, calculated as described in the Methods section. The black line signifies the changing i as HoloMb unfolds.

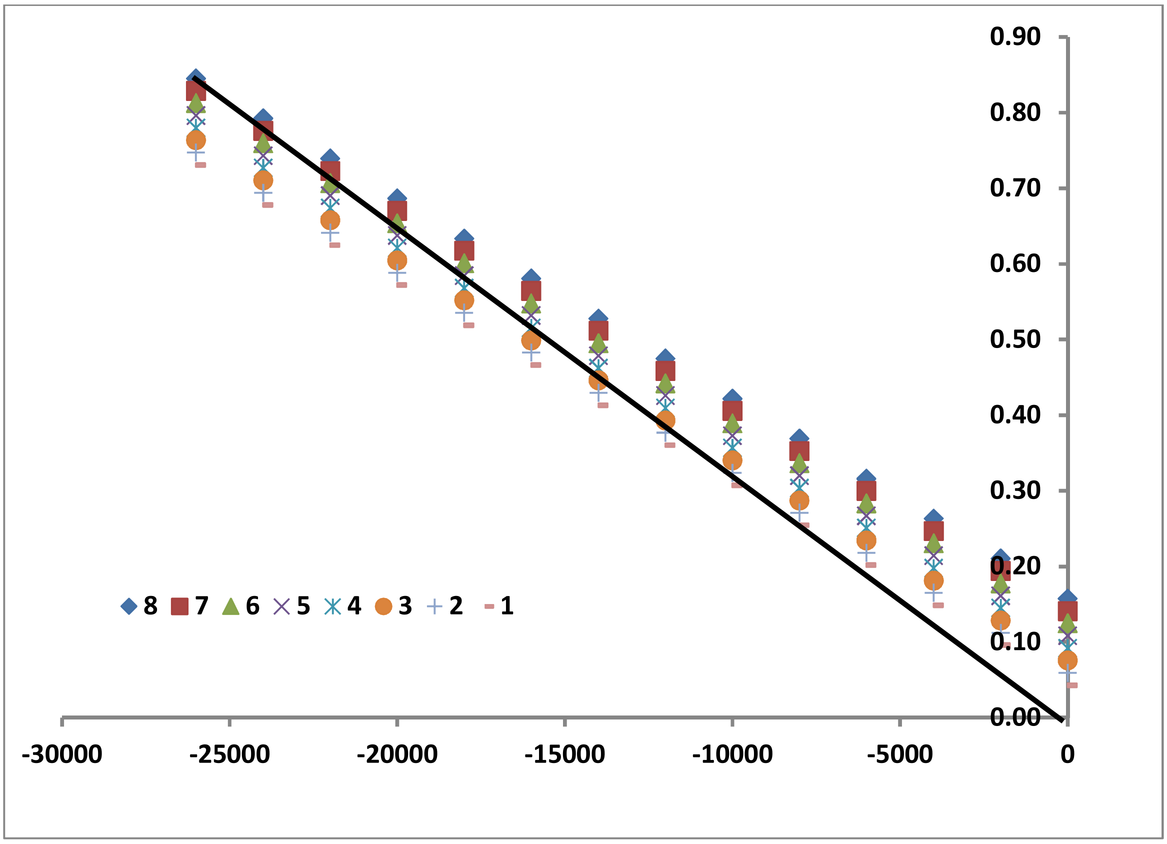

Figure S2. Computed $[\Theta]_{222}$ for Mb structures during unfolding

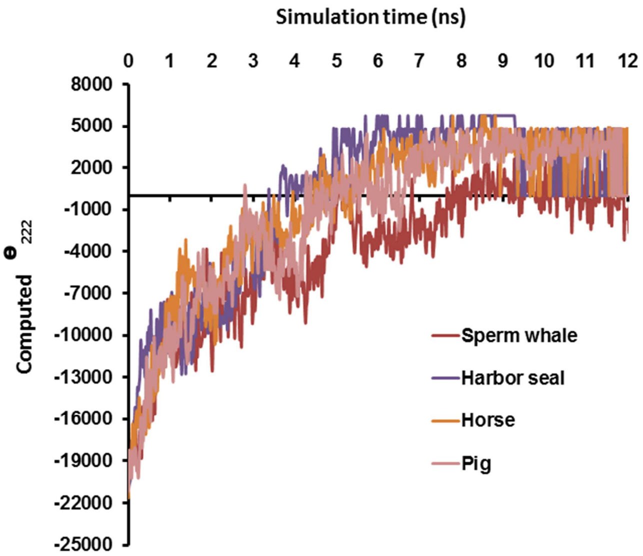

A) $[\Theta]_{helix}$ = 36,800; k = 3.00; $[\Theta]_{RC}$ = 0

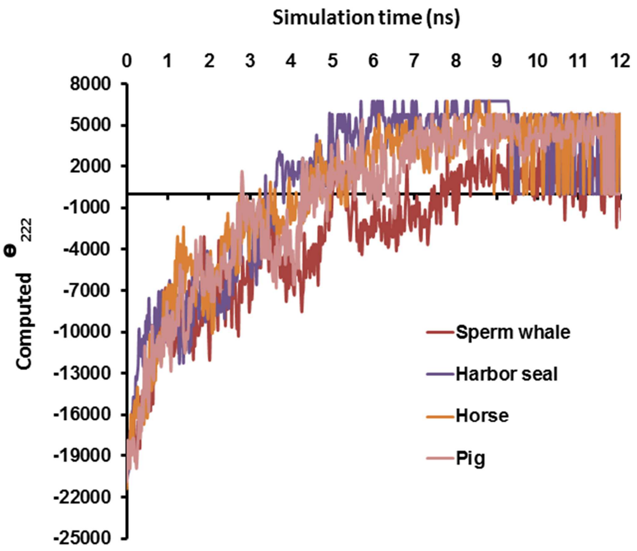

B) $[\Theta]_{helix}$ = 36,800; k = 3.00; $[\Theta]_{RC}$ = 1000

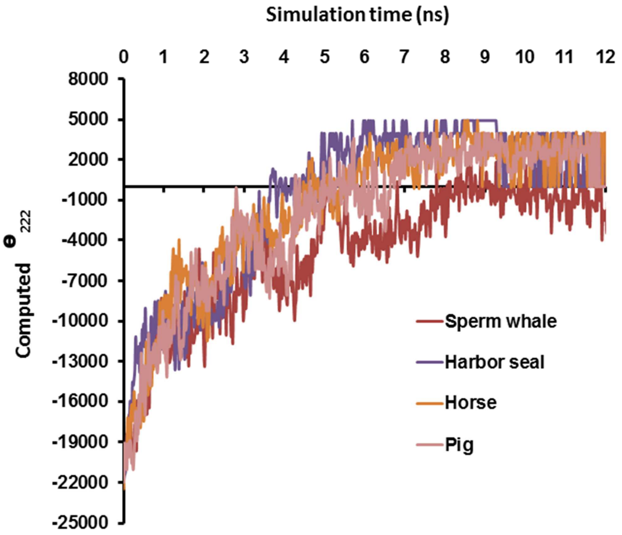

C) $[\Theta]_{helix}$ = 36,800; k = 2.57; $[\Theta]_{RC}$ = 0

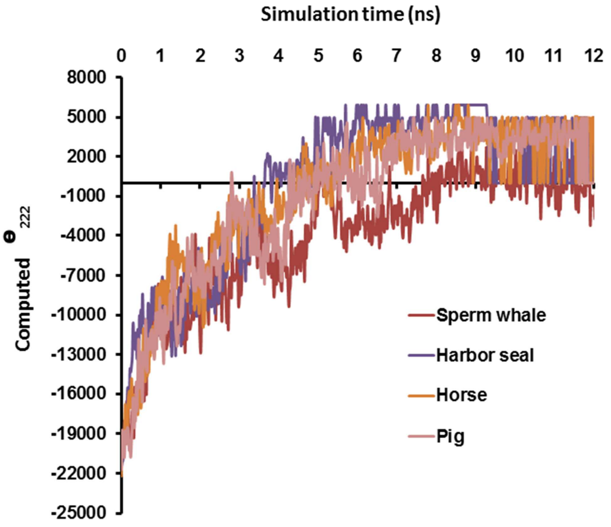

D) $[\Theta]_{helix}$ = 36,800; k = 2.57; $[\Theta]_{RC}$ = 1000

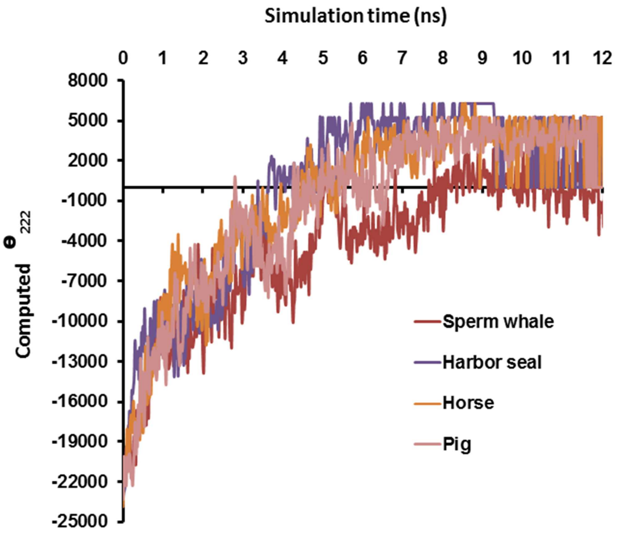

E) $[\Theta]_{helix}$ = 39,500; k = 2.57; $[\Theta]_{RC}$ = 1000

Figure S3. Secondary Structure evolution at 310 K for harbor seal, sperm whale, pig, and horse myoglobins, showing the eight intact helices in blue.

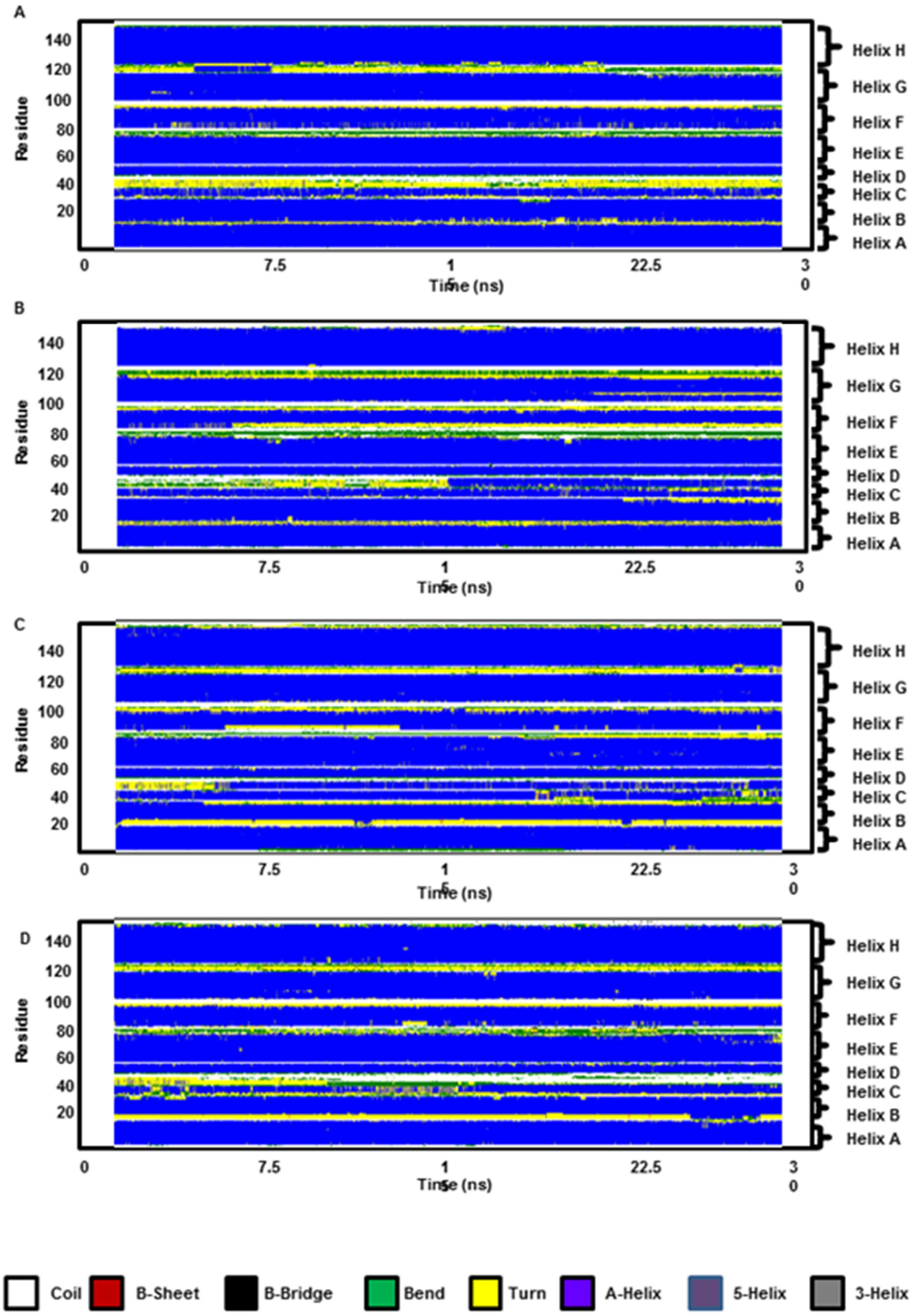

Figure S4. Correlation between experimental crystal structures used for simulation and equilibrated proteins at 310K. From top: Sperm whale, harbor seal, pig, and horse Mb.A: Structural overlays. B: 2D correlation plot.

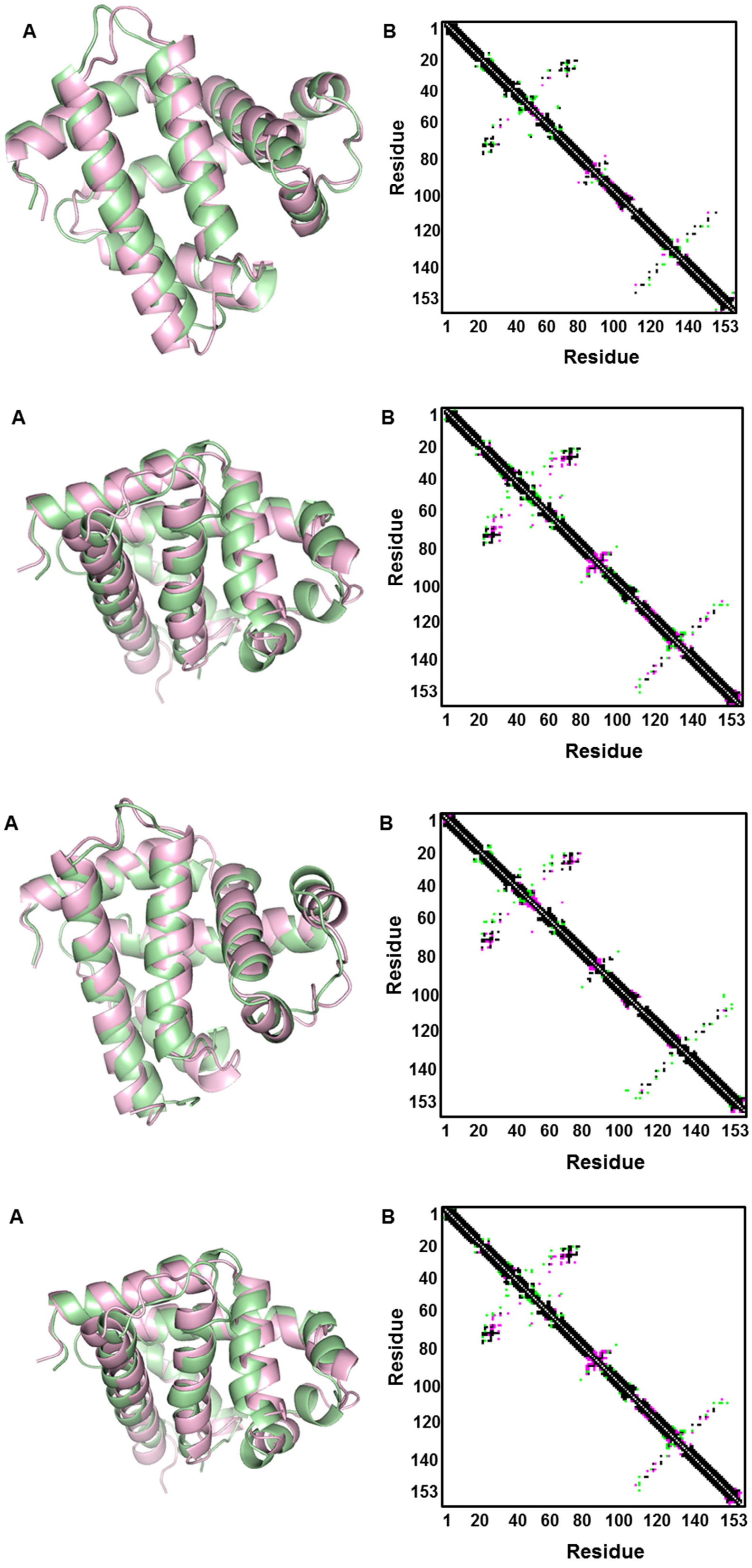

Figure S5. Secondary Structure changes during thermal unfolding for additional simulations: **holo sperm whale Mb at 500K**. Heme is **bound** to His93 during the simulation. Plots were made with the DSSP program.

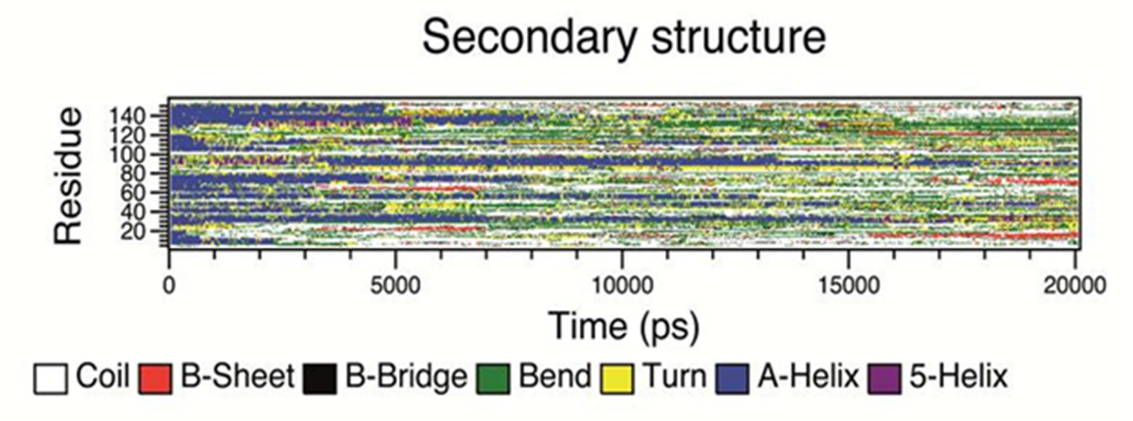

Figure S6. Secondary Structure changes during thermal unfolding for **holo sperm whale Mb at 500K**. Heme is **unbound** to His93 during the simulation. Plots were made with the DSSP program. Two replica simulations.

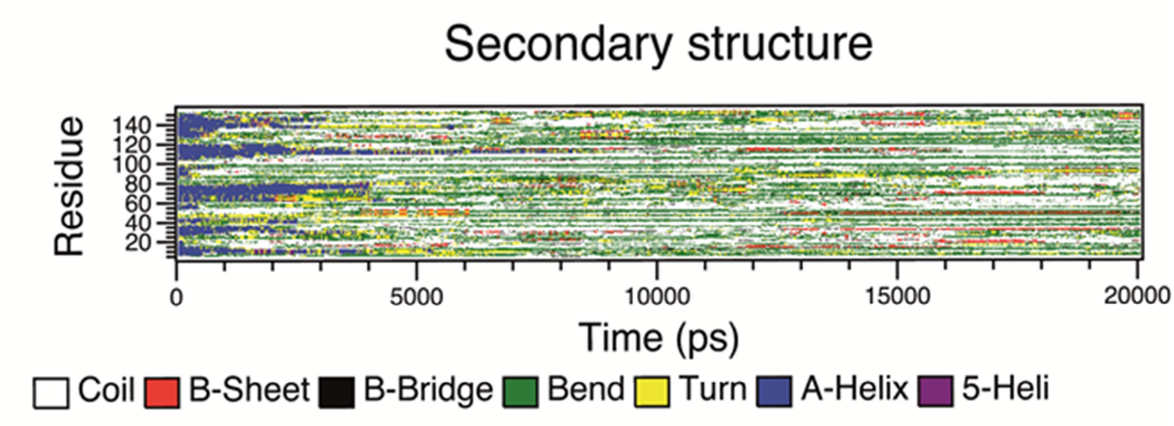

Figure S7. Secondary Structure changes during thermal unfolding for **holo sperm whale Mb at 400K**. Heme is **bound** to His93 during the simulation. Plots were made with the DSSP program.

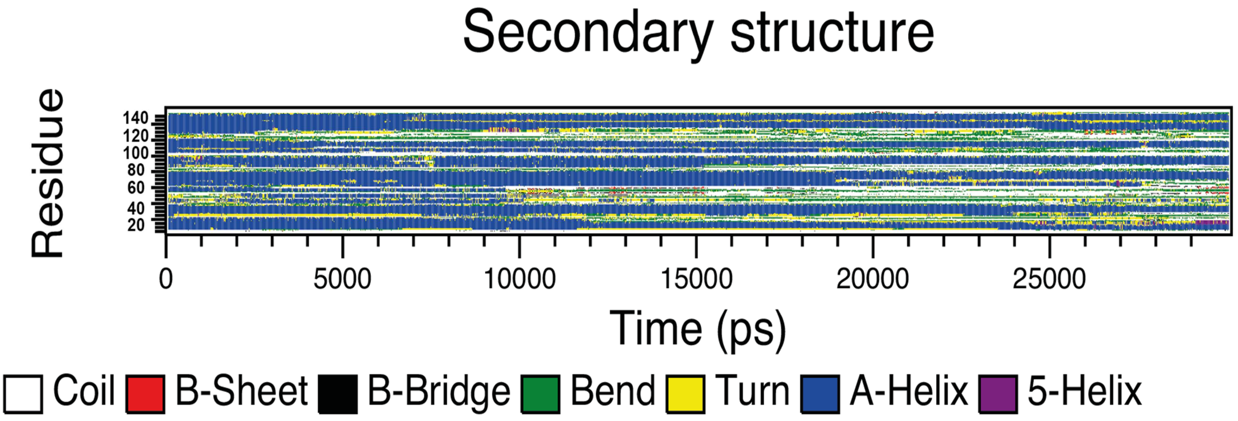

Figure S8. Secondary Structure changes during thermal unfolding for **holo sperm whale Mb at 400K**. Heme is **unbound** to His93 during the simulation. Plots were made with the DSSP program.

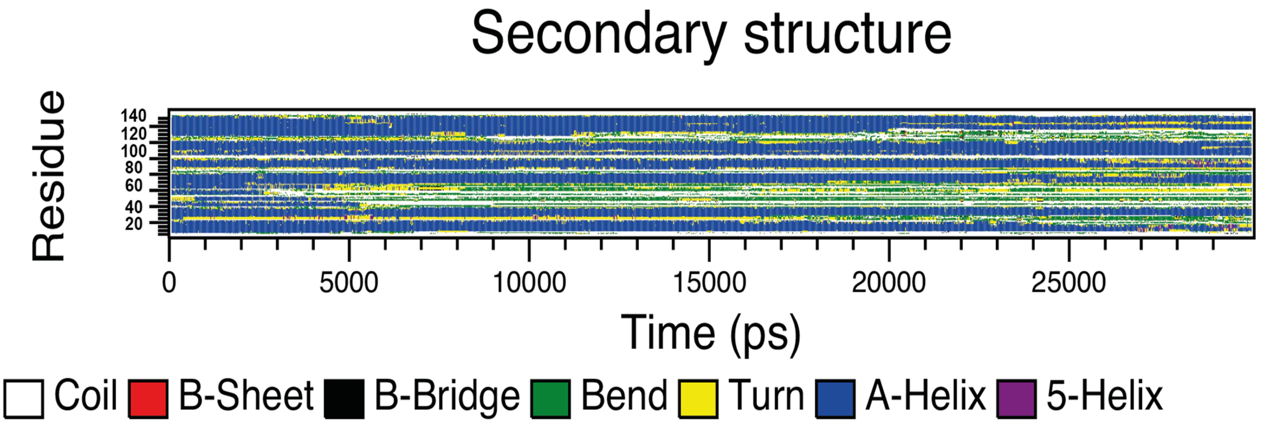

Figure S9. Secondary Structure changes during thermal unfolding for **holo pig Mb at 500K**. Heme is **bound** to His93 during the simulation. Plots were made with the DSSP program.

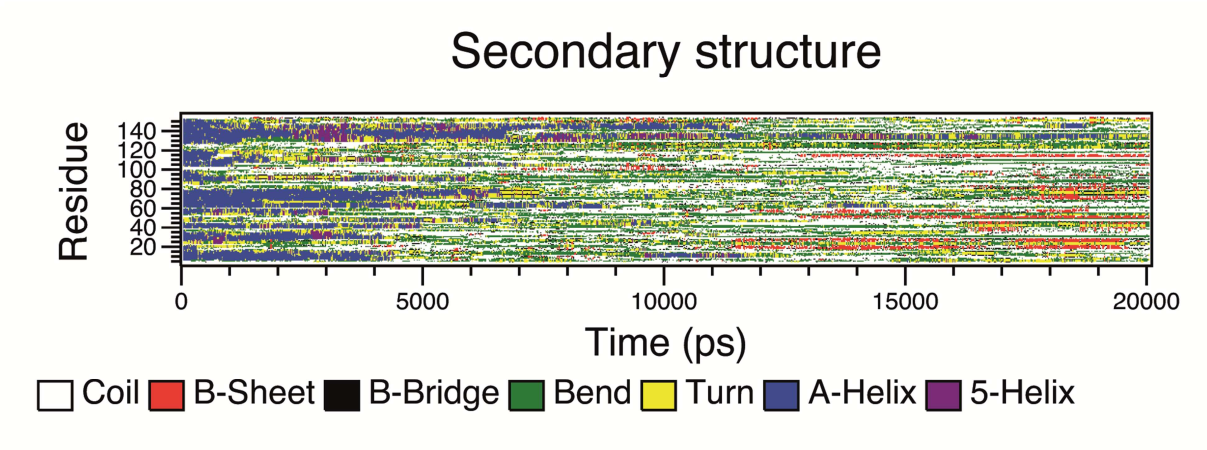

Figure S10. Secondary Structure changes during thermal unfolding for **holo pig Mb at 500K**. Heme is **unbound** to His93 during the simulation. Plots were made with the DSSP program.

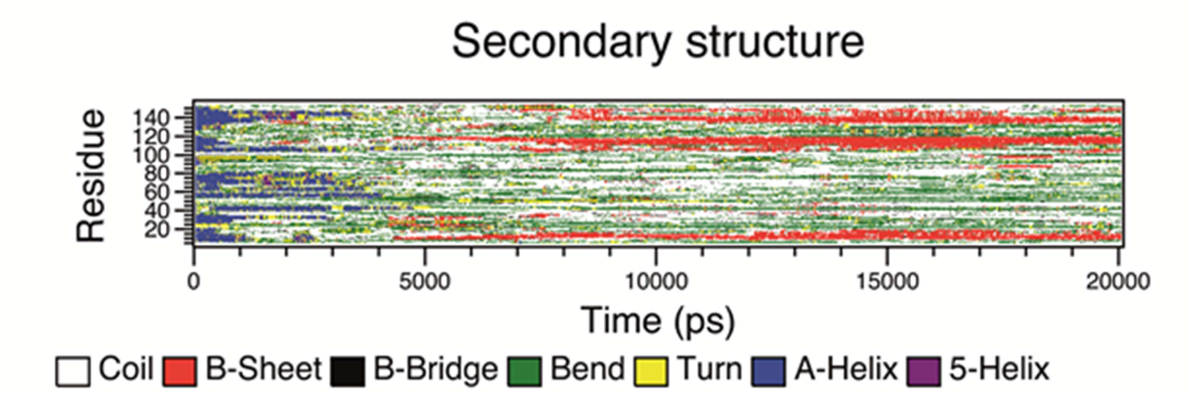

Figure S11. Secondary Structure changes during thermal unfolding for **holo pig Mb at 400K**. Heme is **bound** to His93 during the simulation. Plots were made with the DSSP program.

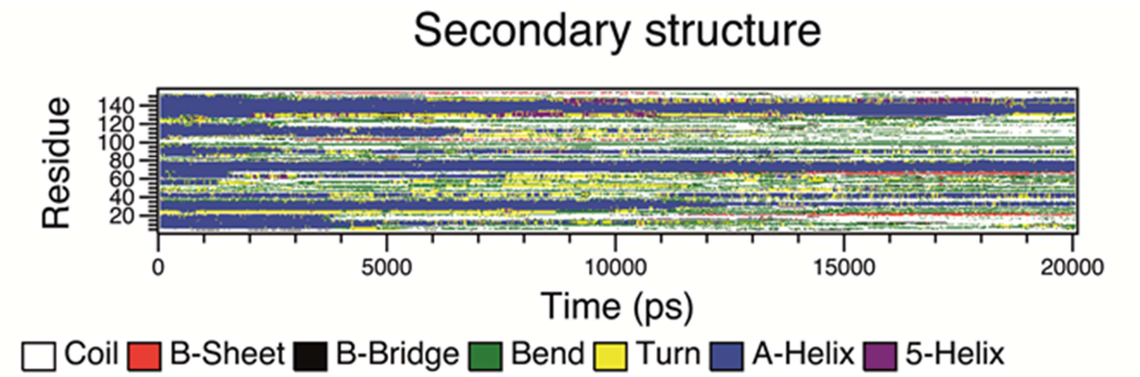

Figure S12. Secondary Structure changes during thermal unfolding for **holo horse Mb at 500K**. Heme is **bound** to His93 during the simulation. Plots were made with the DSSP program.

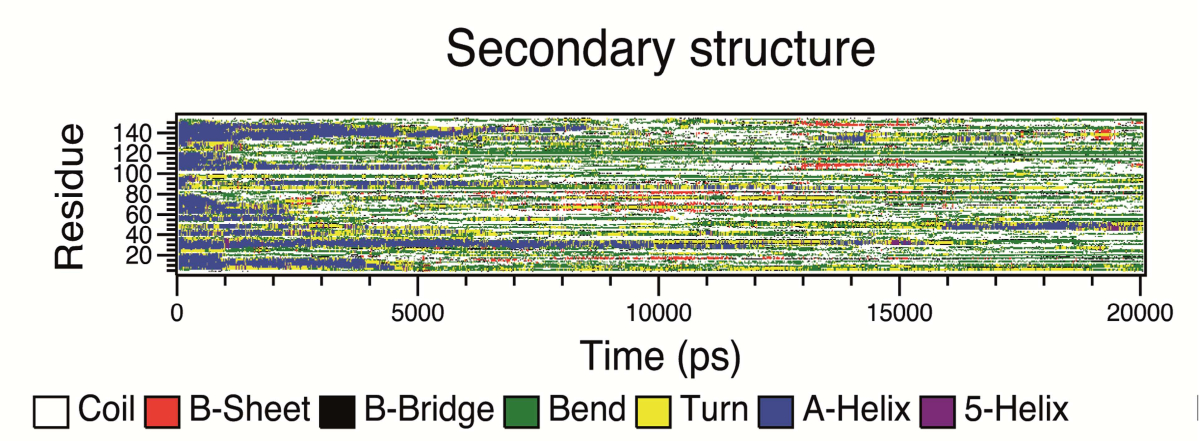

Figure S13. Secondary Structure changes during thermal unfolding for **holo horse Mb at 500K**. Heme is **unbound** to His93 during the simulation. Plots were made with the DSSP program.

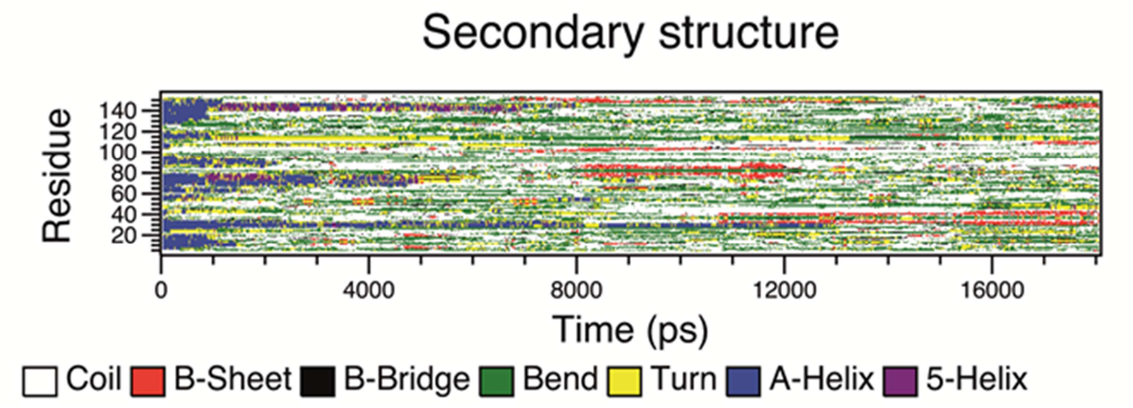

Figure S14. Secondary Structure changes during thermal unfolding for **holo horse Mb at 400K**. Heme is **bound** to His93 during the simulation. Plots were made with the DSSP program.

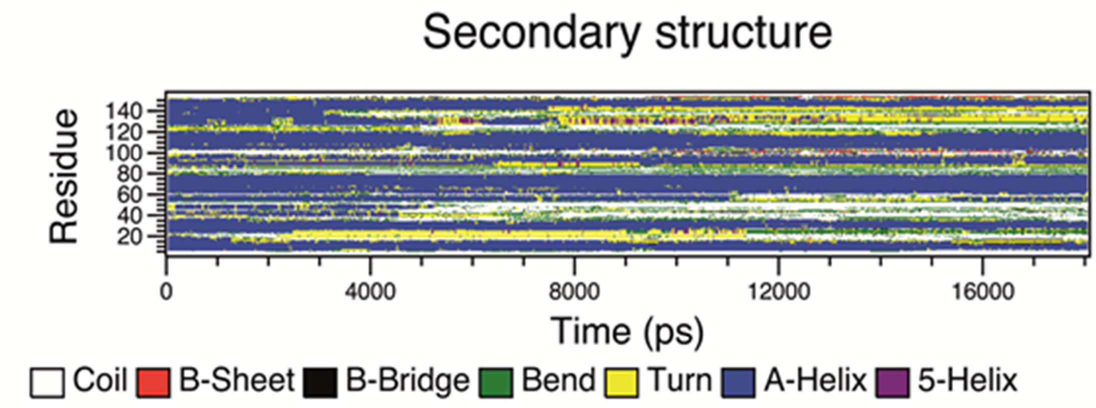

Figure S15. Secondary Structure changes during thermal unfolding for **holo horse Mb at 400K**. Heme is **unbound** to His93 during the simulation. Plots were made with the DSSP program.

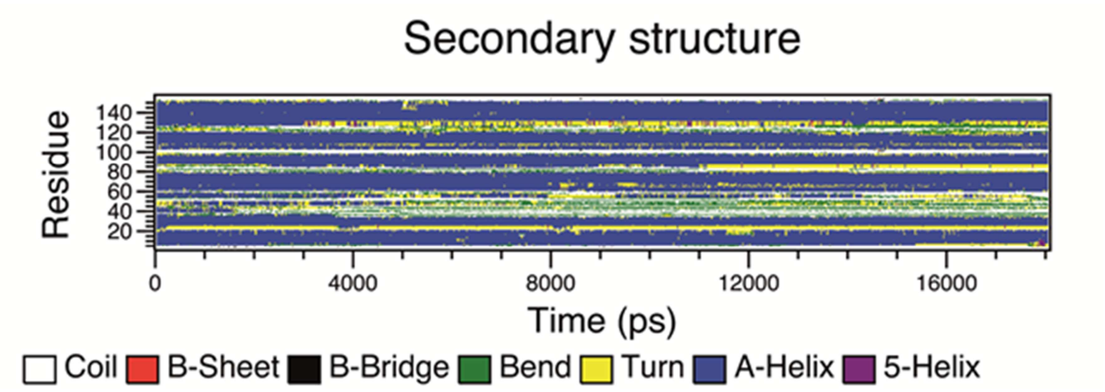

Figure S16. Secondary Structure changes during thermal unfolding for **holo harbor seal Mb at 500K**. Heme is **bound** to His93 during the simulation. Plots were made with the DSSP program.

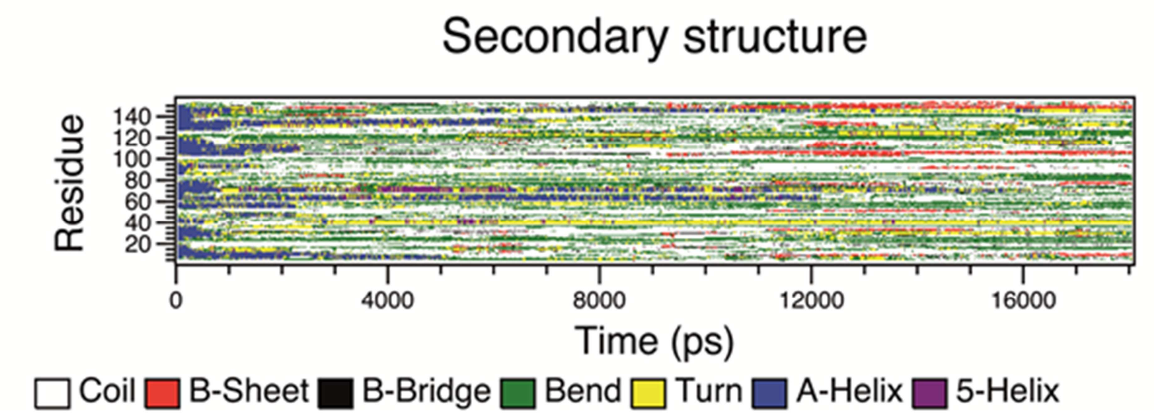

Figure S17. Secondary Structure changes during thermal unfolding for **holo harbor seal Mb at 500K**. Heme is **unbound** to His93 during the simulation. Plots were made with the DSSP program.

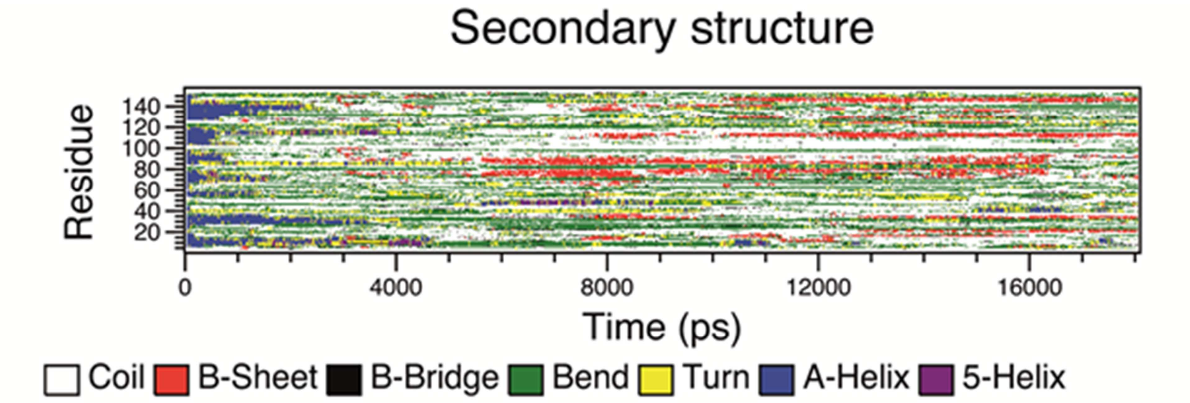

Figure S18. Secondary Structure changes during thermal unfolding for **holo harbor seal Mb at 400K**. Heme is **bound** to His93 during the simulation. Plots were made with the DSSP program.

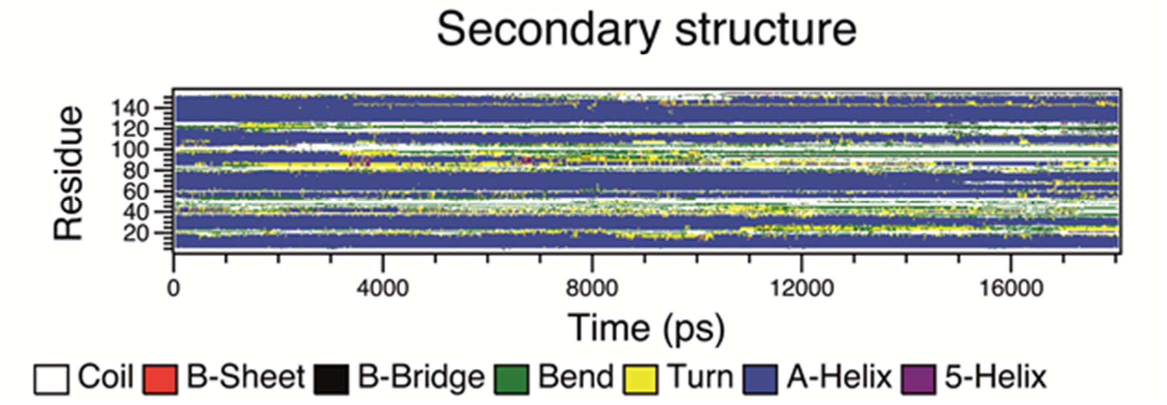

Figure S19. Secondary Structure changes during thermal unfolding for **holo harbor seal Mb at 400K**. Heme is **unbound** to His93 during the simulation. Plots were made with the DSSP program.

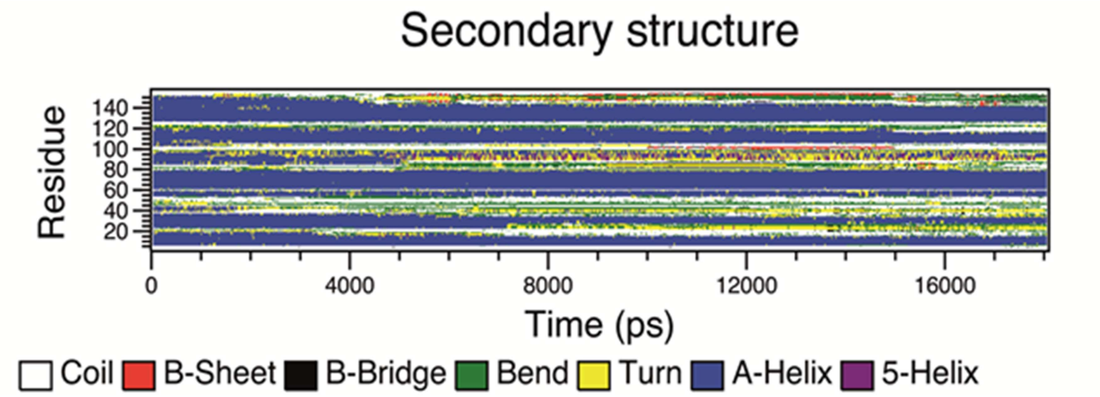

Figure S20. Two-dimensional RMSD matrix for alpha carbon of sperm whale Mb during thermal unfolding at 500K.

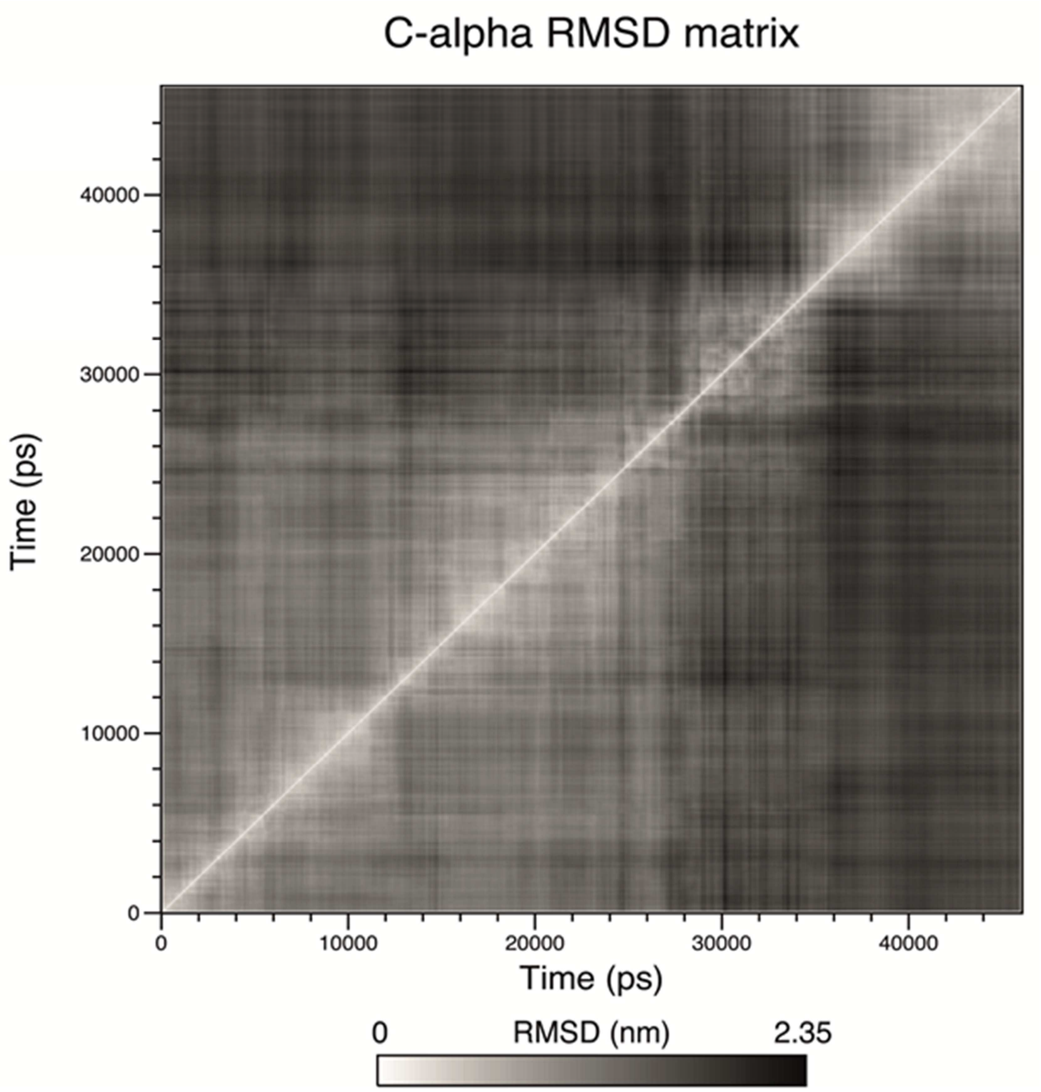

Table S1. Hydrophobic and hydrophilic surface area, SASA, and $\Delta G_{solvation}$ of helix A at T=300K and T=500K.

	**Hydrophobic surface area (nm^2)**							
	**T=300K**		**T=500K**				**Difference (5ns-10ns)**	**Difference (25ns-30ns)**
	**25-30 ns**	**St. dev**	**5-10 ns**	**St. dev**	**25-30 ns**	**St. dev**		
SW	8,95	0,56	7,82	0,88	7,82	0,88	1,12	-0,32
HSEAL	7,61	0,55	10,05	1,02	8,58	0,96	-2,43	-0,47
PIG	7,39	0,61	9,99	1,16	9,63	0,91	-2,60	-0,54
HORSE	7,09	0,61	8,64	0,82	8,28	0,73	-1,55	-0,21
	**Hydrophilic surface area (nm^2)**							
	**T=300K**		**T=500K**				**Difference (5ns-10ns)**	**Difference (25ns-30ns)**
	**25-30 ns**	**St. dev**	**5-10 ns**	**St. dev**	**25-30 ns**	**St. dev**		
SW	4,10	0,33	2,88	0,46	2,88	0,46	1,23	-0,13
HSEAL	4,88	0,31	5,28	0,64	4,13	0,79	-0,41	-0,33
PIG	4,86	0,33	5,18	0,74	5,10	0,65	-0,32	-0,41
HORSE	5,13	0,36	5,18	0,58	4,95	0,65	-0,05	-0,22
	**SASA (nm^2)**							
	**T=300K**		**T=500K**				**Difference (5ns-10ns)**	**Difference (25ns-30ns)**
	**25-30 ns**	**St. dev**	**5-10 ns**	**St. dev**	**25-30 ns**	**St. dev**		
SW	13,05	0,67	10,70	1,20	10,70	1,20	2,35	-0,53
HSEAL	12,49	0,66	15,33	1,32	12,72	1,55	-2,84	-0,66
PIG	12,25	0,76	15,16	1,64	14,73	1,32	-2,92	-0,88
HORSE	12,22	0,75	13,82	1,13	13,22	1,12	-1,60	-0,39
	**ΔG solvation (kJ/mol)**							
	**T=300K**		**T=500K**				**Difference (5ns-10ns)**	**Difference (25ns-30ns)**
	**25-30 ns**	**St. dev**	**5-10 ns**	**St. dev**	**25-30 ns**	**St. dev**		
SW	50,04	4,07	44,02	6,07	44,02	6,07	6,02	-2,01
HSEAL	40,37	4,12	54,14	7,18	47,09	5,92	-13,76	-3,05
PIG	35,01	4,28	53,88	7,43	53,70	6,25	-18,86	-3,15
HORSE	34,10	4,34	44,90	5,42	42,98	5,37	-10,80	-1,08

Table S2. Hydrophobic and hydrophilic surface area, SASA, and $\Delta G_{solvation}$ of helix B at T=300K and T=500K.

	**Hydrophobic surface area (nm^2)**							
	**T=300K**		**T=500K**				**Difference (5ns-10ns)**	**Difference (25ns-30ns)**
	**25-30 ns**	**St. dev**	**5-10 ns**	**St. dev**	**25-30 ns**	**St. dev**		
SW	6,03	0,55	8,87	1,16	8,87	1,16	-2,84	-0,61
HSEAL	5,61	0,52	8,75	0,72	7,28	0,63	-3,14	-0,20
PIG	5,63	0,61	7,47	0,81	9,08	0,97	-1,85	-0,20
HORSE	5,79	0,51	6,52	0,80	6,47	0,80	-0,73	-0,29
	**Hydrophilic surface area (nm^2)**							
	**T=300K**		**T=500K**				**Difference (5ns-10ns)**	**Difference (25ns-30ns)**
	**25-30 ns**	**St. dev**	**5-10 ns**	**St. dev**	**25-30 ns**	**St. dev**		
SW	3,68	0,36	4,54	0,68	4,54	0,68	-0,86	-0,32
HSEAL	3,16	0,33	4,58	0,68	3,43	0,43	-1,42	-0,36
PIG	3,82	0,38	4,61	0,72	4,88	0,69	-0,80	-0,33
HORSE	3,43	0,32	4,04	0,55	2,96	0,50	-0,61	-0,23
	**SASA (nm^2)**							
	**T=300K**		**T=500K**				**Difference (5ns-10ns)**	**Difference (25ns-30ns)**
	**25-30 ns**	**St. dev**	**5-10 ns**	**St. dev**	**25-30 ns**	**St. dev**		
SW	9,71	0,74	13,41	1,68	13,41	1,68	-3,70	-0,94
HSEAL	8,78	0,67	13,33	1,19	10,71	0,84	-4,55	-0,52
PIG	9,44	0,75	12,08	1,34	13,96	1,31	-2,64	-0,59
HORSE	9,22	0,66	10,56	1,15	9,44	1,13	-1,34	-0,49
	**ΔG solvation (kJ/mol)**							
	**T=300K**		**T=500K**				**Difference (5ns-10ns)**	**Difference (25ns-30ns)**
	**25-30 ns**	**St. dev**	**5-10 ns**	**St. dev**	**25-30 ns**	**St. dev**		
SW	33,05	4,05	54,17	8,67	54,17	8,67	-21,11	-4,61
HSEAL	31,53	3,90	52,41	5,07	42,91	4,56	-20,88	-1,18
PIG	34,03	4,49	47,62	5,81	55,43	6,84	-13,59	-1,32
HORSE	37,83	3,55	42,73	5,37	43,52	5,34	-4,90	-1,82

Table S3. Hydrophobic and hydrophilic surface area, SASA, and $\Delta G_{solvation}$ of helix C at T=300K and T=500K.

	**Hydrophobic surface area (nm^2)**							
	**T=300K**		**T=500K**				**Difference (5ns-10ns)**	**Difference (25ns-30ns)**
	**25-30 ns**	**St. dev**	**5-10 ns**	**St. dev**	**25-30 ns**	**St. dev**		
SW	3,32	0,33	3,85	0,60	3,85	0,60	-0,53	-0,27
HSEAL	3,52	0,30	3,75	0,44	3,74	0,44	-0,23	-0,15
PIG	3,21	0,31	4,03	0,57	3,83	0,64	-0,82	-0,26
HORSE	3,49	0,30	3,33	0,49	4,07	0,42	0,15	-0,19
	**Hydrophilic surface area (nm^2)**							
	**T=300K**		**T=500K**				**Difference (5ns-10ns)**	**Difference (25ns-30ns)**
	**25-30 ns**	**St. dev**	**5-10 ns**	**St. dev**	**25-30 ns**	**St. dev**		
SW	2,32	0,22	2,57	0,43	2,57	0,43	-0,25	-0,22
HSEAL	2,13	0,20	2,05	0,30	1,70	0,39	0,07	-0,10
PIG	1,95	0,18	2,14	0,39	2,28	0,32	-0,19	-0,21
HORSE	2,25	0,25	2,37	0,43	2,15	0,38	-0,12	-0,17
	**SASA (nm^2)**							
	**T=300K**		**T=500K**				**Difference (5ns-10ns)**	**Difference (25ns-30ns)**
	**25-30 ns**	**St. dev**	**5-10 ns**	**St. dev**	**25-30 ns**	**St. dev**		
SW	5,65	0,41	6,42	0,91	6,42	0,91	-0,78	-0,50
HSEAL	5,65	0,36	5,81	0,62	5,44	0,70	-0,16	-0,26
PIG	5,15	0,40	6,16	0,86	6,11	0,75	-1,01	-0,46
HORSE	5,74	0,41	5,70	0,78	6,21	0,63	0,04	-0,37
	**ΔG solvation (kJ/mol)**							
	**T=300K**		**T=500K**				**Difference (5ns-10ns)**	**Difference (25ns-30ns)**
	**25-30 ns**	**St. dev**	**5-10 ns**	**St. dev**	**25-30 ns**	**St. dev**		
SW	15,95	2,78	19,17	4,03	19,17	4,03	-3,22	-1,25
HSEAL	17,76	2,27	20,16	3,38	19,85	3,12	-2,40	-1,11
PIG	12,02	2,31	18,50	3,81	16,91	4,51	-6,48	-1,50
HORSE	15,83	2,51	17,33	3,29	20,98	3,37	-1,50	-0,78

Table S4. Hydrophobic and hydrophilic surface area, SASA, and $\Delta G_{solvation}$ of helix D at T=300K and T=500K.

	**Hydrophobic surface area (nm^2)**							
	**T=300K**		**T=500K**				**Difference (5ns-10ns)**	**Difference (25ns-30ns)**
	**25-30 ns**	**St. dev**	**5-10 ns**	**St. dev**	**25-30 ns**	**St. dev**		
SW	4,39	0,29	3,85	0,68	3,85	0,68	0,53	-0,39
HSEAL	2,67	0,27	3,06	0,46	3,11	0,50	-0,40	-0,19
PIG	3,48	0,34	3,71	0,45	3,62	0,48	-0,23	-0,11
HORSE	3,90	0,34	4,35	0,71	4,27	0,68	-0,45	-0,37
	**Hydrophilic surface area (nm^2)**							
	**T=300K**		**T=500K**				**Difference (5ns-10ns)**	**Difference (25ns-30ns)**
	**25-30 ns**	**St. dev**	**5-10 ns**	**St. dev**	**25-30 ns**	**St. dev**		
SW	3,24	0,27	3,24	0,68	3,24	0,68	0,00	-0,41
HSEAL	3,96	0,35	4,27	0,60	3,75	0,60	-0,31	-0,26
PIG	4,13	0,28	3,55	0,50	3,77	0,49	0,58	-0,22
HORSE	3,23	0,25	3,39	0,52	3,62	0,50	-0,16	-0,27
	**SASA (nm^2)**							
	**T=300K**		**T=500K**				**Difference (5ns-10ns)**	**Difference (25ns-30ns)**
	**25-30 ns**	**St. dev**	**5-10 ns**	**St. dev**	**25-30 ns**	**St. dev**		
SW	7,63	0,41	7,10	1,23	7,10	1,23	0,53	-0,82
HSEAL	6,62	0,45	7,33	0,89	6,86	0,96	-0,71	-0,44
PIG	7,61	0,45	7,26	0,78	7,39	0,80	0,35	-0,33
HORSE	7,13	0,45	7,74	1,09	7,89	0,99	-0,61	-0,64
	**ΔG solvation (kJ/mol)**							
	**T=300K**		**T=500K**				**Difference (5ns-10ns)**	**Difference (25ns-30ns)**
	**25-30 ns**	**St. dev**	**5-10 ns**	**St. dev**	**25-30 ns**	**St. dev**		
SW	19,01	2,54	17,37	4,27	17,37	4,27	1,64	-1,73
HSEAL	12,33	2,25	15,33	3,31	16,77	3,46	-2,99	-1,06
PIG	8,68	2,78	13,18	3,43	13,04	3,46	-4,50	-0,65
HORSE	14,64	2,52	18,97	5,17	17,71	4,76	-4,33	-2,65

Table S5. Hydrophobic and hydrophilic surface area, SASA, and $\Delta G_{solvation}$ of helix E at T=300K and T=500K.

	**Hydrophobic surface area (nm²)**							
	**T=300K**		**T=500K**				**Difference (5ns-10ns)**	**Difference (25ns-30ns)**
	**25-30 ns**	**St. dev**	**5-10 ns**	**St. dev**	**25-30 ns**	**St. dev**		
SW	9,05	0,61	11,22	0,81	11,22	0,81	-2,17	-0,20
HSEAL	8,35	0,49	9,14	1,27	8,34	0,76	-0,79	-0,78
PIG	8,51	0,65	7,82	0,96	9,08	0,84	0,69	-0,31
HORSE	9,52	0,63	10,60	0,90	11,20	0,80	-1,09	-0,27
	**Hydrophilic surface area (nm²)**							
	**T=300K**		**T=500K**				**Difference (5ns-10ns)**	**Difference (25ns-30ns)**
	**25-30 ns**	**St. dev**	**5-10 ns**	**St. dev**	**25-30 ns**	**St. dev**		
SW	2,88	0,26	4,18	0,55	4,18	0,55	-1,30	-0,29
HSEAL	4,80	0,35	5,55	1,17	4,54	0,57	-0,75	-0,82
PIG	3,19	0,28	2,57	0,57	3,08	0,44	0,61	-0,29
HORSE	2,79	0,27	3,55	0,55	3,93	0,68	-0,76	-0,28
	**SASA (nm²)**							
	**T=300K**		**T=500K**				**Difference (5ns-10ns)**	**Difference (25ns-30ns)**
	**25-30 ns**	**St. dev**	**5-10 ns**	**St. dev**	**25-30 ns**	**St. dev**		
SW	11,93	0,75	15,40	1,10	15,40	1,10	-3,47	-0,36
HSEAL	13,15	0,66	14,69	2,31	12,88	1,08	-1,55	-1,65
PIG	11,69	0,76	10,39	1,40	12,16	1,09	1,30	-0,63
HORSE	12,31	0,74	14,16	1,27	15,13	1,25	-1,85	-0,53
	**ΔG solvation (kJ/mol)**							
	**T=300K**		**T=500K**				**Difference (5ns-10ns)**	**Difference (25ns-30ns)**
	**25-30 ns**	**St. dev**	**5-10 ns**	**St. dev**	**25-30 ns**	**St. dev**		
SW	42,00	4,46	59,21	5,91	59,21	5,91	-17,21	-1,45
HSEAL	43,51	3,81	50,47	8,07	47,31	5,35	-6,96	-4,26
PIG	36,01	4,65	36,22	6,44	42,85	6,39	-0,21	-1,79
HORSE	42,70	4,65	54,81	5,72	56,69	5,59	-12,11	-1,06

Table S6. Hydrophobic and hydrophilic surface area, SASA, and $\Delta G_{solvation}$ of helix F at T=300K and T=500K.

	**Hydrophobic surface area (nm^2)**							
	**T=300K**		**T=500K**				**Difference (5ns-10ns)**	**Difference (25ns-30ns)**
	**25-30 ns**	**St. dev**	**5-10 ns**	**St. dev**	**25-30 ns**	**St. dev**		
SW	5,44	0,39	5,04	0,60	5,04	0,60	0,40	-0,21
HSEAL	4,74	0,39	4,20	0,51	5,84	0,76	0,54	-0,12
PIG	4,61	0,39	6,07	0,73	4,89	0,68	-1,46	-0,34
HORSE	4,69	0,43	4,43	0,87	5,75	0,79	0,26	-0,43
	**Hydrophilic surface area (nm^2)**							
	**T=300K**		**T=500K**				**Difference (5ns-10ns)**	**Difference (25ns-30ns)**
	**25-30 ns**	**St. dev**	**5-10 ns**	**St. dev**	**25-30 ns**	**St. dev**		
SW	2,10	0,24	2,04	0,33	2,04	0,33	0,06	-0,10
HSEAL	2,16	0,28	2,14	0,33	3,36	0,54	0,01	-0,05
PIG	2,28	0,28	3,84	0,62	2,74	0,68	-1,55	-0,34
HORSE	2,23	0,36	2,17	0,71	3,30	0,60	0,07	-0,35
	**SASA (nm^2)**							
	**T=300K**		**T=500K**				**Difference (5ns-10ns)**	**Difference (25ns-30ns)**
	**25-30 ns**	**St. dev**	**5-10 ns**	**St. dev**	**25-30 ns**	**St. dev**		
SW	7,53	0,46	7,08	0,80	7,08	0,80	0,45	-0,34
HSEAL	6,89	0,51	6,34	0,68	9,20	1,14	0,55	-0,18
PIG	6,90	0,53	9,91	1,22	7,63	1,22	-3,01	-0,69
HORSE	6,92	0,66	6,60	1,49	9,05	1,25	0,32	-0,83
	**ΔG solvation (kJ/mol)**							
	**T=300K**		**T=500K**				**Difference (5ns-10ns)**	**Difference (25ns-30ns)**
	**25-30 ns**	**St. dev**	**5-10 ns**	**St. dev**	**25-30 ns**	**St. dev**		
SW	26,79	2,86	24,67	4,09	24,67	4,09	2,12	-1,24
HSEAL	27,57	2,98	25,36	3,52	34,33	4,94	2,21	-0,54
PIG	26,44	2,85	37,16	5,04	30,16	4,79	-10,73	-2,18
HORSE	27,27	3,46	26,80	6,11	35,31	5,39	0,46	-2,66

Table S7. Hydrophobic and hydrophilic surface area, SASA, and $\Delta G_{solvation}$ of helix G at T=300K and T=500K.

	**Hydrophobic surface area (nm^2)**							
	**T=300K**		**T=500K**				**Difference (5ns-10ns)**	**Difference (25ns-30ns)**
	**25-30 ns**	**St. dev**	**5-10 ns**	**St. dev**	**25-30 ns**	**St. dev**		
SW	8,26	0,64	10,57	0,83	10,57	0,83	-2,31	-0,19
HSEAL	9,13	0,74	11,46	0,97	11,63	0,82	-2,33	-0,23
PIG	8,24	0,66	10,45	1,15	11,67	0,89	-2,21	-0,49
HORSE	7,39	0,57	10,73	0,86	11,10	0,96	-3,33	-0,29
	**Hydrophilic surface area (nm^2)**							
	**T=300K**		**T=500K**				**Difference (5ns-10ns)**	**Difference (25ns-30ns)**
	**25-30 ns**	**St. dev**	**5-10 ns**	**St. dev**	**25-30 ns**	**St. dev**		
SW	5,03	0,35	6,03	0,78	6,03	0,78	-1,00	-0,43
HSEAL	4,00	0,31	4,31	0,62	4,49	0,52	-0,31	-0,31
PIG	5,44	0,38	5,05	0,81	6,12	0,64	0,39	-0,43
HORSE	5,19	0,29	5,52	0,53	5,31	0,52	-0,33	-0,24
	**SASA (nm^2)**							
	**T=300K**		**T=500K**				**Difference (5ns-10ns)**	**Difference (25ns-30ns)**
	**25-30 ns**	**St. dev**	**5-10 ns**	**St. dev**	**25-30 ns**	**St. dev**		
SW	13,29	0,76	16,60	1,26	16,60	1,26	-3,31	-0,50
HSEAL	13,13	0,90	15,77	1,35	16,12	1,16	-2,64	-0,46
PIG	13,68	0,82	15,50	1,71	17,79	1,29	-1,82	-0,89
HORSE	12,58	0,66	16,25	1,19	16,41	1,22	-3,67	-0,53
	**ΔG solvation (kJ/mol)**							
	**T=300K**		**T=500K**				**Difference (5ns-10ns)**	**Difference (25ns-30ns)**
	**25-30 ns**	**St. dev**	**5-10 ns**	**St. dev**	**25-30 ns**	**St. dev**		
SW	51,40	4,58	66,67	5,47	66,67	5,47	-15,27	-0,89
HSEAL	52,02	5,50	66,65	6,41	66,59	6,08	-14,63	-0,91
PIG	45,38	4,63	62,58	8,02	69,33	5,92	-17,21	-3,39
HORSE	43,63	3,94	66,91	6,09	67,54	6,27	-23,28	-2,15

Table S8. Hydrophobic and hydrophilic surface area, SASA, and $\Delta G_{solvation}$ of helix G at T=300K and T=500K.

	Hydrophobic surface area (nm^2)							
	**T=300K**		**T=500K**				**Difference (5ns-10ns)**	**Difference (25ns-30ns)**
	**25-30 ns**	**St. dev**	**5-10 ns**	**St. dev**	**25-30 ns**	**St. dev**		
SW	11,08	0,68	14,91	0,98	14,91	0,98	-3,83	-0,31
HSEAL	11,61	0,66	14,15	1,05	14,34	0,80	-2,54	-0,39
PIG	10,83	0,58	12,68	1,15	13,12	1,08	-1,85	-0,57
HORSE	10,50	0,70	11,75	1,03	12,36	1,20	-1,26	-0,33
	**Hydrophilic surface area (nm^2)**							
	**T=300K**		**T=500K**				**Difference (5ns-10ns)**	**Difference (25ns-30ns)**
	**25-30 ns**	**St. dev**	**5-10 ns**	**St. dev**	**25-30 ns**	**St. dev**		
SW	5,68	0,38	6,78	0,85	6,78	0,85	-1,10	-0,47
HSEAL	6,53	0,39	7,68	0,72	7,65	0,63	-1,16	-0,32
PIG	6,89	0,54	7,86	0,88	7,63	0,77	-0,97	-0,34
HORSE	6,72	0,45	6,94	0,66	6,83	0,83	-0,22	-0,21
	**SASA (nm^2)**							
	**T=300K**		**T=500K**				**Difference (5ns-10ns)**	**Difference (25ns-30ns)**
	**25-30 ns**	**St. dev**	**5-10 ns**	**St. dev**	**25-30 ns**	**St. dev**		
SW	16,75	0,81	21,69	1,55	21,69	1,55	-4,93	-0,74
HSEAL	18,13	0,78	21,83	1,50	21,99	1,15	-3,70	-0,72
PIG	17,72	0,85	20,54	1,76	20,74	1,61	-2,82	-0,91
HORSE	17,22	0,92	18,70	1,34	19,19	1,79	-1,48	-0,43
	**ΔG solvation (kJ/mol)**							
	**T=300K**		**T=500K**				**Difference (5ns-10ns)**	**Difference (25ns-30ns)**
	**25-30 ns**	**St. dev**	**5-10 ns**	**St. dev**	**25-30 ns**	**St. dev**		
SW	50,82	5,03	81,92	6,78	81,92	6,78	-31,10	-1,75
HSEAL	55,39	4,95	73,34	7,64	73,42	5,79	-17,95	-2,70
PIG	51,66	4,73	64,95	7,70	69,60	6,94	-13,30	-2,97
HORSE	51,27	4,91	63,90	6,84	69,46	7,89	-12,63	-1,93

Table S9. Calculated heme loss times from MD simulation of holomyoglobins at 500K, using the criteria explained in the Methods section.

Species	Heme loss time (ns)	
	400K	500K, replicate 1, replicate 2
Sperm whale	>26	~3.0, 3.7
Pig	>16	~1.0, 1.8
Horse	>16	~1.0, 1.6
Harbor seal	>16	~1.7, 3.0

Figure S21. The 2D plot of Number of main-chain-mainc-hain hydrogen bonds vs. RMSD as reaction coordinates in the simulations of holo PigMb at 500K. Three distinct clusters are observed. Each point is reported at 20ps intervals.

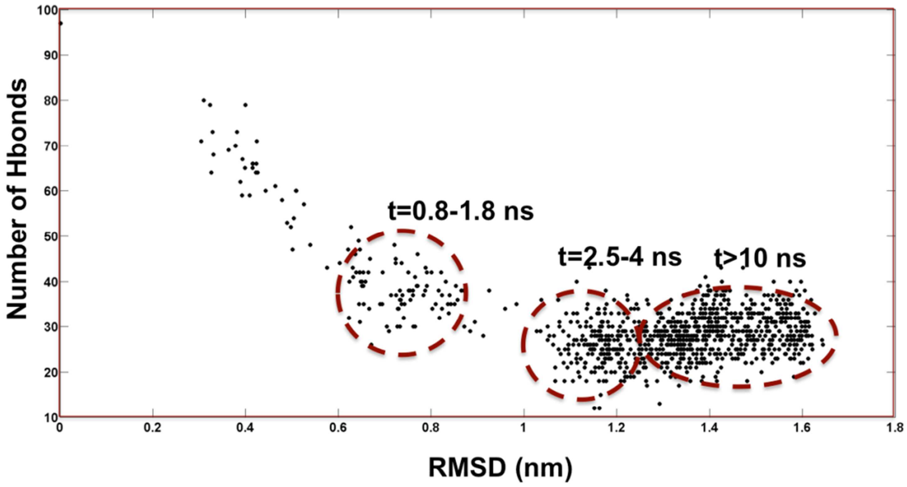

Figure S22. The 2D plot of Number of main-chain-mainc-hain hydrogen bonds vs. RMSD as reaction coordinates in the simulations of holo HorseMb at 500K. Three distinct clusters are observed. Each point is reported at 20ps intervals.

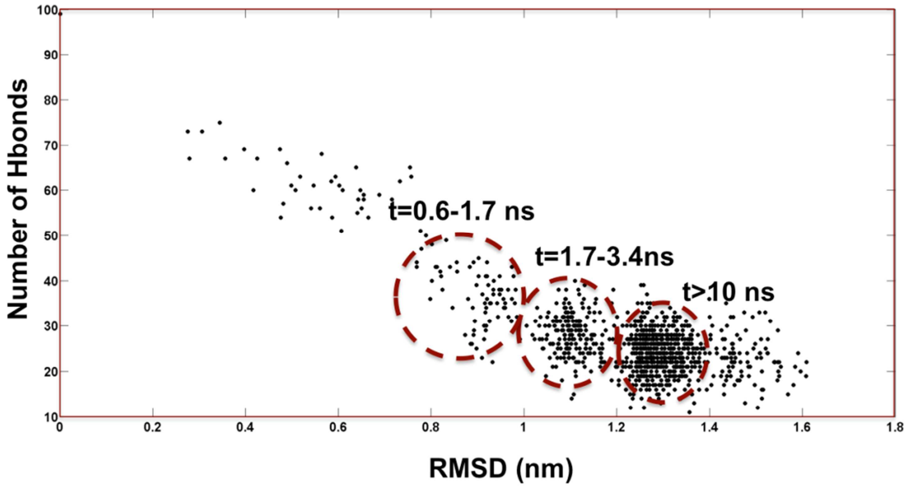

Figure S23. The 2D plot of Number of main-chain-mainc-hain hydrogen bonds vs. RMSD as reaction coordinates in the simulations of holo haror seal Mb at 500K. Three distinct clusters are observed. Each point is reported at 20ps intervals.

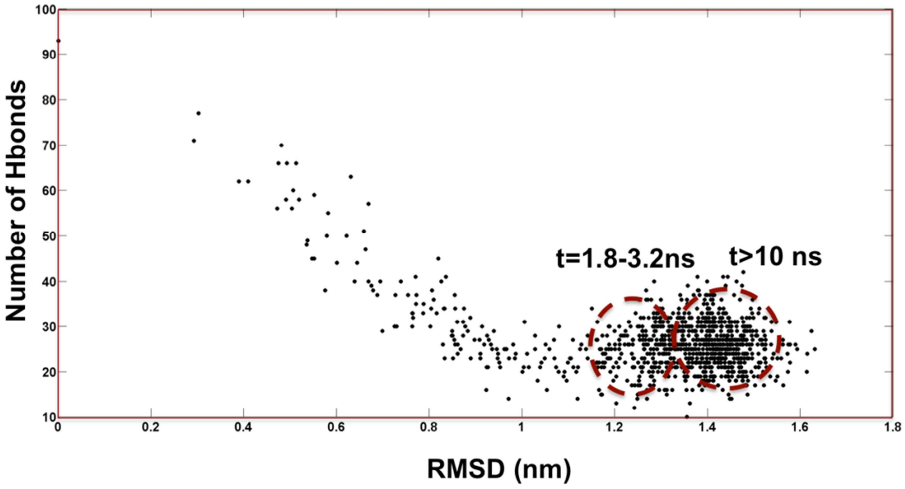

Figure S24. The 2D plot of Number of main-chain-mainc-hain hydrogen bonds vs. RMSD as reaction coordinates in the simulations of sperm whale seal Mb at 500K. Three distinct clusters are observed. Each point is reported at 20ps intervals.

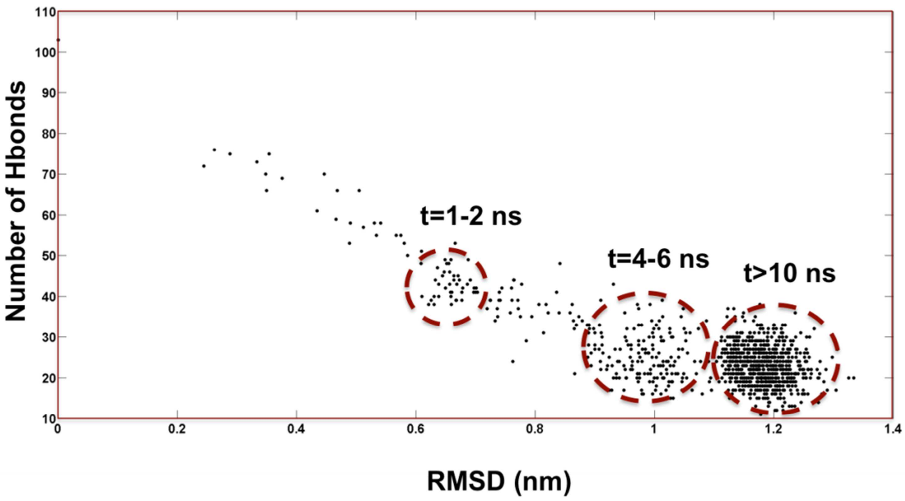

Supplement: File S1 — contains information about the relation between helix character and ellipticity (Figure S1); figures showing the sensitivity to variation in parameters (Figure S2); Secondary Structure types at 310 K for all four proteins (Figure S3); correlation of structures to experimental structures (Figure S4); Time evolution of secondary structures (Figures S5–S19); 2D RMSD matrix for alpha carbon (Figure S20); Key average properties of the simulated proteins separated into helices (Tables S1–S8); Timings of heme loss from simulations at 500 K, first and second replicate (Table S9); clustering analysis for all four proteins (Figures S21–S24). (PDF) [file pone.0080308.s001.pdf]
